# Exploring endoperoxides as a new entry for the synthesis of branched azasugars

**DOI:** 10.3762/bjoc.13.63

**Published:** 2017-04-03

**Authors:** Svenja Domeyer, Mark Bjerregaard, Henrik Johansson, Daniel Sejer Pedersen

**Affiliations:** 1 Department of Drug Design and Pharmacology, University of Copenhagen, Jagtvej 162, 2100 Copenhagen, Denmark

**Keywords:** azasugar, carbohydrate, cycloaddition, endoperoxide, photochemistry

## Abstract

A new class of nitrogen-containing endoperoxides were synthesised by a photochemical [4 + 2]-cycloaddition between a diene and singlet oxygen. The endoperoxides were dihydroxylated and protected to provide a series of endoperoxide building blocks for organic synthesis, with potential use as precursors for the synthesis of branched azasugars. Preliminary exploration of the chemistry of these building blocks provided access to a variety of derivatives including tetrahydrofurans, epoxides and protected amino-tetraols.

## Introduction

**Scheme 1:**
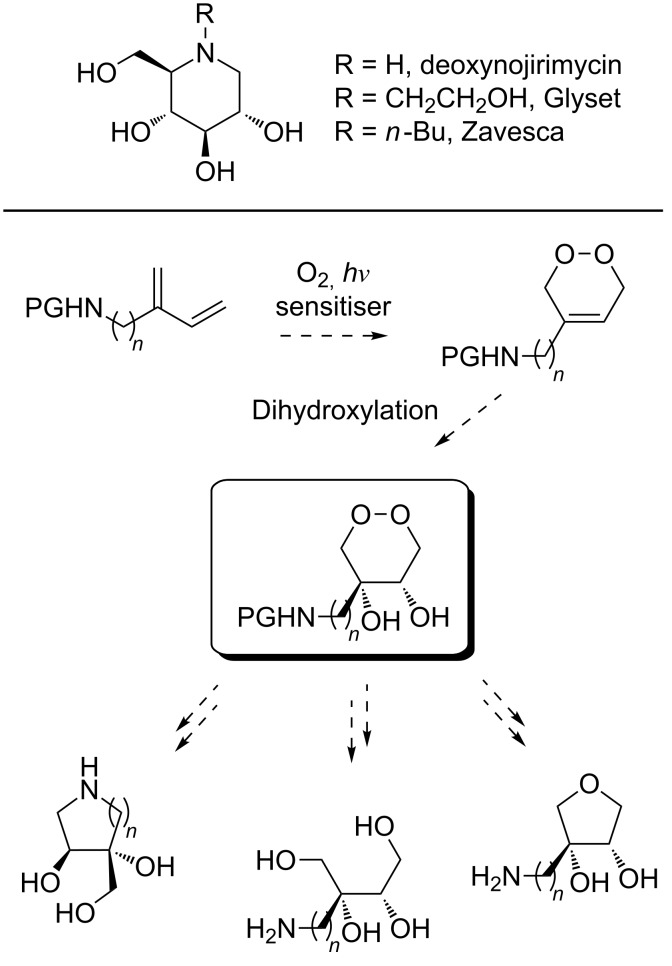
Top: The natural product deoxynojirimycin and two analogues and marketed drugs Glyset and Zavesca. Bottom: Our synthetic strategy aimed to explore substituted endoperoxides (e.g. dihydroxylated endoperoxide, box) as key intermediates for the divergent synthesis of novel azasugars. PG = protection group.

Azasugars are small organic compounds that can mimic carbohydrates or their hydrolysis transition states as well as having many other interesting properties. They are found in nature as pyrrolidines, piperidines, indolizidines, pyrrolizidines or nortropane alkaloids with a variety of ring substituents, typically hydroxy groups, carboxylic acids and amides [1]. The ability of azasugars, such as the natural product deoxynojirimycin, to inhibit the activity of a wide range of enzymes has attracted attention for their potential use as drug candidates [2–3]. Azasugars in clinical use today include Glyset, a licensed drug for treatment of diabetes type II, and Zavesca, which is used in treatment of type I Gaucher’s disease and Niemann Pick type C disease (Scheme 1).

Historically, azasugars were most commonly synthesised from available carbohydrate starting materials, a strategy that is still extensively employed today [4–5]. However, a variety of alternative synthetic strategies have since been developed, including asymmetric and chemoenzymatic methods [2,6–8]. Substituted endoperoxides have been applied to the synthesis of substituted cyclopropanes, furans and carbohydrates [9–12]. Inspired by the work of Robinson and co-workers [10–12] we hypothesised that endoperoxides might provide a new entry to branched azasugars with novel stereochemistry and substitution patterns (Scheme 1). Herein we wish to report our preliminary results in synthesising nitrogen containing endoperoxides as a potential new source for the divergent synthesis of azasugars.

## Results and Discussion

**Scheme 2:**
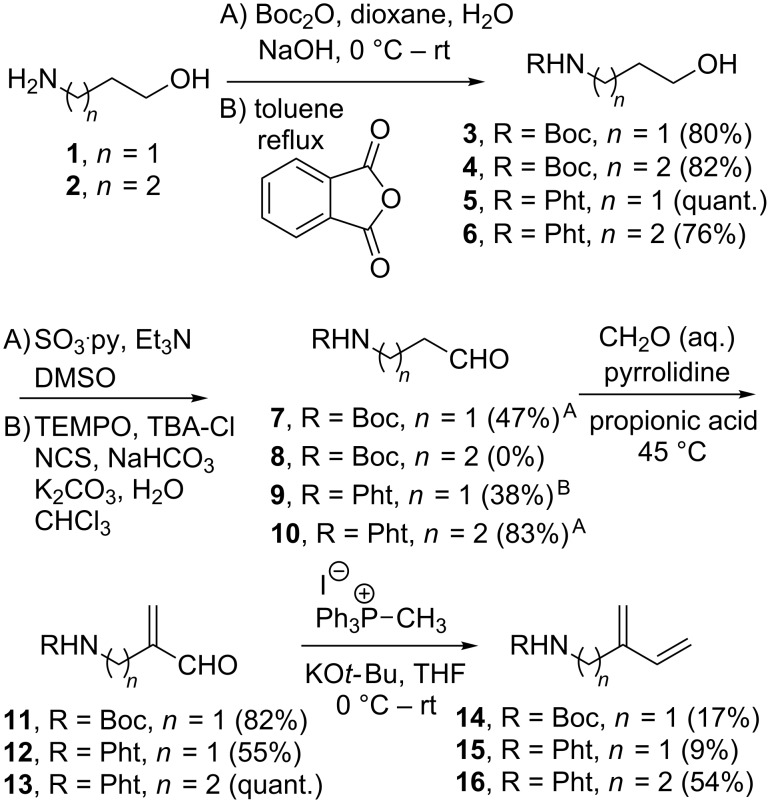
Synthesis of Boc- and Pht-protected diene substrates for endoperoxide synthesis. TBA-Cl = tetrabutylammonium chloride, NCS = *N*-chlorosuccinimide.

We set out to synthesise two diene substrates for a photochemical [4 + 2]-cycloaddition with singlet oxygen to provide two different endoperoxides (Scheme 1, *n* = 1–2). Based on literature precedent Boc-protection was utilised for the amino group (Scheme 2) [13–14]. Moreover, the phthalimide-protected compounds were synthesised as UV active analogues that would facilitate analysis.

Aminoalcohols **1** and **2** were Boc- and Pht-protected under standard conditions to provide alcohols **3–6** in good yield. Oxidation of alcohol **3** to aldehyde **7** was achieved in moderate yield using standard Parikh–Doering oxidation conditions [15]. However, when the same conditions were applied to alcohol **4** it resulted in a complex mixture. Other oxidation procedures, including TPAP [16], Dess–Martin periodinane [17] and TEMPO [18] were attempted but in all instances complex mixtures were obtained and none of the desired aldehyde **8** was observed. Presumably the problems encountered with compound **4** originates from the ability of the carbamate nitrogen to form a five-membered hydroxypyrrolidine that can undergo further oxidation [19]. Oxidation of alcohol **5** to give aldehyde **9** was accomplished in a low yield using TEMPO, whereas oxidation of **6** using the Parikh–Doering method gave a good yield of aldehyde **10**. Next, aldehydes **7, 9** and **10** were subjected to a Mannich reaction using the method by Erkkilä and Pihko to give the α,β-unsaturated aldehydes **11–13** in good yields [20]. The final step of the diene synthesis was a Wittig reaction with methyltriphenylphosphonium iodide to provide **14–16**. Compound **14** was found to be volatile and thus was only isolated in a poor yield. For diene **15** the low yield was largely due to complications with removing the triphenylphosphine oxide side product. However, at this stage sufficient material of dienes **14–16** was available to proceed to the critical cycloaddition and dihydroxylation steps and thus no further optimisation was performed.

**Scheme 3:**
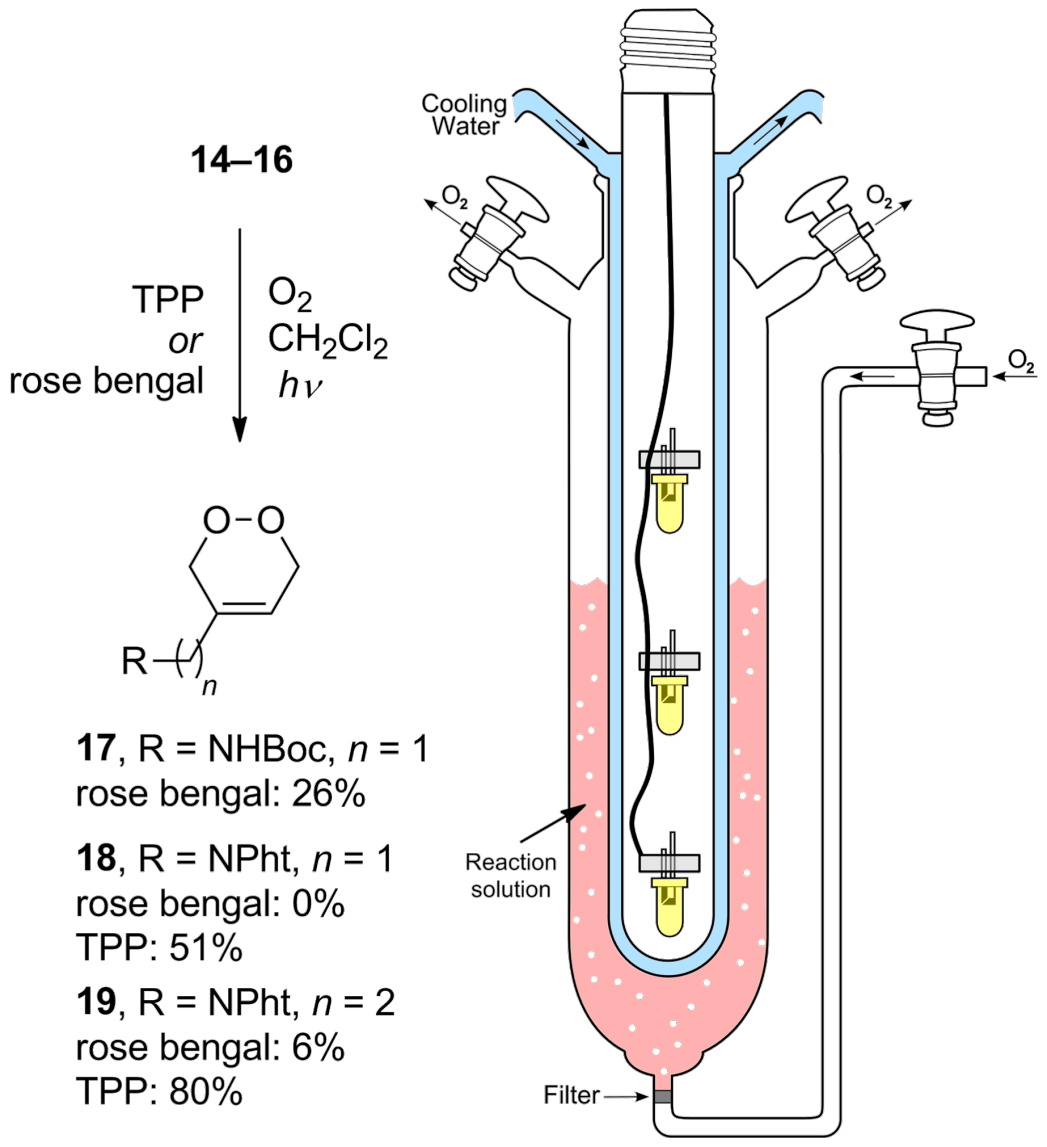
Synthesis of endoperoxides **17–19** by [4 + 2]-cycloaddition of dienes **14–16** with singlet oxygen. The photochemical reactor was modified with a gas inlet fitted with a filter at the bottom of the reactor, and the original mercury lamp was replaced with three 100 W halogen bulbs.

The cycloaddition reaction of dienes **14–16** with singlet oxygen was performed in a modified photochemical reactor that had been fitted with a gas inlet tube at the bottom to allow bubbling of oxygen through the reaction mixture whilst simultaneously cooling and irradiating (Scheme 3). During the synthesis of diene **14** we found that it was volatile and thus we anticipated problems in the cycloaddition due to the reaction set up. As anticipated when the reaction was performed with the sensitizer rose bengal the diene was observed to quickly disappear from the system and only a low yield of endoperoxide **17** was obtained. Due to the many problems encountered with the Boc-protection strategy it was abandoned at this stage and we turned our attention to the phthalimide-protected dienes **15**,**16**. When the same reaction was performed with rose bengal on diene **16** a very low yield of endoperoxide **19** was obtained and most starting material was recovered (78%) even after prolonged reaction time (40 h). However, the reaction outcome was greatly improved by exchanging the sensitizer with tetraphenylporphyrin (TPP) to give a good yield of the desired endoperoxide **19**. Likewise, diene **15** was converted to the corresponding endoperoxide **18** in good yield using TPP, whereas no product was obtained using rose bengal.

**Scheme 4:**
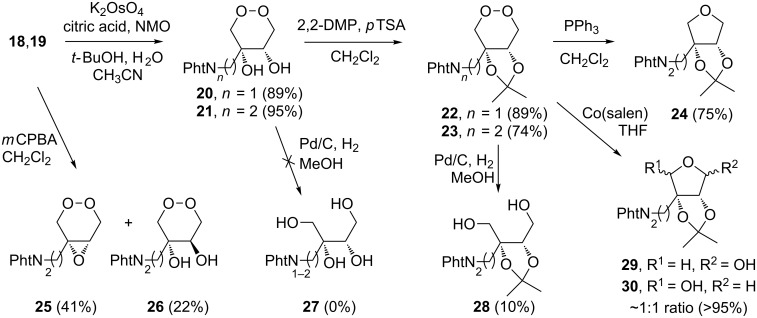
Dihydroxylation and protection of endoperoxides **18** and **19** to provide novel building blocks **20–23** for azasugar synthesis. Preliminary exploration of the chemistry of endoperoxides **18–23** was encouraging and gave access to a variety of derivatives.

With endoperoxides **18** and **19** in hand the stage was set for the central dihydroxylation reaction that would give access to key intermediates **20** and **21** (Scheme 4). To our delight dihydroxylation with potassium osmate in the presence of citric acid was smooth and gave excellent yields of diols **20** and **21** [11]. Diols **20** and **21** were found to be unstable unless stored at −20 °C. However, protection of the diol moiety as an acetonide to give endoperoxides **22** and **23** rendered the compounds stable at ambient temperature. Compounds **18–23** lend themselves to undergo a wide range of chemical transformations and are therefore valuable building blocks that will allow in-depth exploration of the chemistry of this class of azasugar precursors. Some preliminary experiments were performed to assess the possibilities that these building blocks grant.

Endperoxide **19** was epoxidized under standard conditions to provide the desired epoxide **25** and the anti-diol **26** as a by-product. Likely, diol **26** is formed by ring-opening of the epoxide by water present in the *m*CPBA and the reaction could be optimised by performing the reaction under anhydrous conditions. Attempts at cleaving the endoperoxide bond of **20** and **21** by catalytic hydrogenation resulted in rapid decomposition. However, when the same conditions were applied to acetonide-protected endoperoxide **23** the desired diol **28** was obtained, albeit in low yield, with the lactol isomers **29** and **30** as the major product (45%). It is somewhat surprising that the lactols were formed under these conditions and it is anticipated that the reaction conditions could be optimised to favour the desired diol product. Ring contraction of acetonide-protected endoperoxide **23** by treatment with triphenylphosphine provided ready access to tetrahydrofuran **24** in good yield [21]. Finally, treatment of endoperoxide **23** with Co(salen) gave ready access to the two lactol isomers **29**,**30** in quantitative yield (≈1:1 ratio of isomers) [10,22–23]. Lactol isomers **29**,**30** could potentially serve as precursors for the formation of substituted piperidines via reductive amination or be oxidised to yield lactones or lactam derivatives.

## Conclusion

Herein, we report the development of a synthetic strategy using [4 + 2]-cycloaddition reactions to yield endoperoxides **18–23** that contain a protected primary amine. Such intermediates serve as useful new building blocks for organic synthesis. Preliminary exploration of the chemistry of these compounds was encouraging and gave access to a variety of novel protected azasugar precursors. We conclude that the choice of amine and dihydroxy protecting groups can have an impact on the success of the endoperoxide synthesis and its thermal stability, and that employing phthalimide and acetonide groups for *N-* and *O-*protection, respectively, allow for a range of chemical transformations to be carried out. We are in the process of optimising and exploring the chemistry of these building blocks and the results will be reported in due course.

## Supporting Information

File 1 Experimental procedures for all compounds; ^1^H and ^13^C NMR spectra for novel compounds **13**, **16**, **18**–**28**.
