# Supplementary material for: Exploring endoperoxides as a new entry for the synthesis of branched azasugars
Source: Beilstein J Org Chem. 2017 Apr 3;13:644–7. doi: 10.3762/bjoc.13.63 (PMC5389192; doi:10.3762/bjoc.13.63)
Supplement: File 1 — Experimental procedures for all compounds; 1H and 13C NMR spectra for novel compounds 13, 16, 18–28. [file Beilstein_J_Org_Chem-13-644-s001.pdf]

Supporting Information

for

Exploring endoperoxides as a new entry for the synthesis of branched azasugars

Svenja Domeyer, Mark Bjerregaard, Henrik Johansson[1] and Daniel Sejer Pedersen*

Address: Department of Drug Design and Pharmacology, University of Copenhagen, Jagtvej 162, 2100 Copenhagen, Denmark

Email: Daniel Sejer Pedersen - daniel.pedersen@sund.ku.dk

*Corresponding author

Experimental procedures for all compounds; ^{1}H and ^{13}C NMR spectra for novel compounds 13, 16, 18–28

Table of contents

General experimental……………………………………………………………………...S2

Experimental procedures………………………………………………………….…..…S4

NMR spectra…………………………………………………………………………….S27

References……………………………………………………………………………....S39

General

All purchased chemicals were used without further purification. Anhydrous reactions were performed in oven- or flame-dried glassware under argon. Solvents were of chromatography grade and were dried using a SD Water solvent purification system (CH_2Cl_2, THF) or with 3 Å molecular sieves. Sulfate buffer was prepared by dissolving 1.876 mol Na_2SO_4 in 0.625 mol H_2SO_4 to a total volume of 2500 ml. Flash column chromatography was carried out using silica gel 60 (40 – 63 µm). TLC was carried out on Merck pre-coated silica gel 60 F_{254} plates and compounds were visualised using UV light (254 nm), ninhydrin, potassium permanganate or anisaldehyde stains. Retention factor (R_f) values were reported and rounded to the nearest 0.05. HPLC was recorded on a Dionex 3000 Ultimate instrument. A Gemini-NX 3u RP C18 column (250 × 4.6 mm) with UV detection at 210, 254 and 280 nm was used. Mobile phase (MP) A: 0.1% TFA , 100% H_2O (v/v). MP B: 0.1% TFA, 10% H2O, 90% CH_3CN (v/v/v). Flow rate: 1 ml/min. Gradient: 0–15 min: 0–100% MP B, 15–20 min 100% MP B. Retention times (t_R) are reported in minutes. IR was recorded on a Perkin-Elmer Spectrum One fourier transform FTIR spectrometer with a universal attenuated total reflectance (ATR) device and signals (v_{max}) are reported in wavenumbers (cm^{-1}). Nuclear magnetic resonance spectroscopy (NMR) spectra were recorded on a 400 or 600 MHz Bruker instrument and analysed using MestReNova software. Chemical shifts are reported in ppm (δ) using the solvent ($CDCl_3$ or MeOD) as reference. The recorded signals are denoted by the following abbreviations or combinations thereof: br (broad), m (multiplet), s (singlet), d (doublet), t (triplet), q (quartet), quint (quintet) and assessed from chemical shifts, coupling constants, COSY, HSQC and HMBC experiments. Coupling constants (*J*) are reported in Hertz (Hz) and rounded to the nearest 0.5 Hz. Low resolution mass spectrometry (LRMS) was recorded on a Bruker Esquire 3000 plus instrument

connected to an Agilent 1200 HPLC system using an electrospray ionization (ESI) mass detector. Bruker Daltonics DataAnalysis Version 3.3 was used for data analysis. High resolution mass spectrometry (HRMS) was performed in positive ion mode with MALDI ionisation on a Thermo QExactive Orbitrap mass spectrometer equipped with an APSMALDI 10 ion source and operated at mass resolving power 140,000@*m*/*z*200. DHB was used as matrix and lock-mass for internal mass calibration.

The photochemical reactor used herein was made by modification of a standard photochemical reactor with a mercury lamp. A glassblower attached a gas inlet tube with a sintered glass filter to the bottom of the reactor to allow bubbling oxygen gas through the solution. Finally, the mercury lamp from the original system was replaced by three 100 W halogen bulbs from Philips (Capsuleline 100W GY6.35 12V CL 4000H 1CT/10X10F Single-ended low-voltage halogen capsule giving crisp white halogen light).

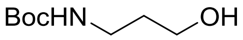

***N*-(*tert*-Butoxycarbonyl)-3-hydroxypropylamine (3)**

3-Aminopropan-1-ol (1.0 g, 13.3 mmol) was suspended in 1,4-dioxane (20 ml). Water (10 ml) and aq. NaOH (1 M, 10 ml) were added, and the mixture was cooled to 0 °C in an ice-water bath. Di-*tert*-butyl dicarbonate (3.2 g, 14.6 mmol) was added, the cooling bath removed and the mixture stirred at ambient temperature. After 5 h additional di-*tert*-butyl dicarbonate (1.2 g, 5.3 mmol) was added. After 18 h the reaction mixture was transferred to a separatory funnel with sulfate buffer (40 ml) and extracted with EtOAc (3 × 30 ml). The combined organic phases were washed with saturated aq. $NaHCO_3$ (90 ml), brine (90 ml), dried (Na_2SO_4) and concentrated in vacuo. Purification by flash column chromatography (40% EtOAc in *n*-heptane, v/v) gave the carbamate **3** (1.86 g, 80%) as a colourless oil.

TLC R_f = 0.35 (40% EtOAc in *n*-heptane, v/v).

IR (neat) v_{max} 3348, 2978, 1684, 1517, 1366, 1252, 1167 cm^{-1}.

^{1}H NMR (600 MHz, $CDCl_3$) δ 4.76 (br s, 1H, OH), 3.69 (q, *J* = 6 Hz, 2H, CH_2OH), 3.32 (q, *J* = 6.5 Hz, 2H, NHCH_2), 2.87 (d, *J* = 7 Hz, 1H, NH), 1.69 (quint, *J* = 6 Hz, 2H, $NHCH_2CH_2$), 1.47 (s, 9H, $C(CH_3)_3$).

^{13}C NMR (151 MHz, $CDCl_3$) δ 157.2 (NCOO), 79.5 ($C(CH_3)_3$), 59.2 (CH_2OH), 36.9 ($NHCH_2$), 32.8 (CH_2CH_2OH), 28.4 (($C(CH_3)_3$).

The analytical data is in agreement with that previously reported [1].

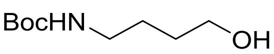

***tert*-Butyl (4-hydroxybutyl)carbamate (4)**

4-Aminobutan-1-ol (1.0 g, 11.2 mmol) was suspended in a mixture of dioxane (20 ml), H_2O (10 ml) and 1 M NaOH (10 ml) and cooled to 0 °C (ice-water bath) with

stirring. Boc_2O (2.69 g, 12.34 mmol) was added and stirring was continued at ambient temperature. After 5.5 h additional Boc_2O (1.08 g, 4.95 mmol) was added and stirring was continued overnight. Next day additional Boc_2O (1.35 g, 6.19 mmol) was added to the suspension. After 1.5 h sulfate buffer (40 ml) was added and the reaction mixture was transferred to a separating funnel and extracted with EtOAc (3 × 30 ml). The combined organic layers were washed with $NaHCO_3$ (90 ml) and brine (90 ml), dried (Na_2SO_4), filtered and concentrated in vacuo. Purification by flash column chromatography (60 % EtOAc in *n*-heptane, v/v) gave carbamate **4** (1.62 g, 82%) as a colourless oil.

TLC R_f = 0.3 (60 % EtOAc in *n*-heptane, v/v).

IR (neat) ν_{max} 3347, 2935, 1686, 1527, 1170 cm^{-1}.

^{1}H NMR (600 MHz; $CDCl_3$): δ 4.59 (br s, 1H, OH), 3.67 (q, J = 6 Hz, 2H, CH_2OH), 3.19-3.13 (m, 2H, CH_2NH), 1.64-1.53 (m, 5H, CH_2CH_2 and NH), 1.44 (s, 9H, Boc).

^{13}C NMR (151 MHz; $CDCl_3$) δ 156.2 (C=O), 79.3 (*C*(CH_3)$_3$), 62.7 (CH_2OH), 40.4 (NCH_2), 29.9 (CH_2), 28.6 (C(*C*H_3)$_3$), 26.8 (CH_2).

The analytical data is in agreement with that reported previously [2].

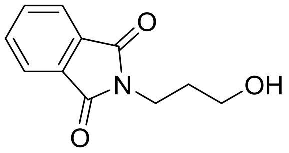

2-(3-Hydroxypropyl)isoindoline-1,3-dione (5)

3-Aminopropan-1-ol (10.0 g, 133.1 mmol) was dissolved in anhydrous toluene (100 ml). Phthalic anhydride (19.7 g, 133.1 mmol) was added and the reaction mixture was heated to 125 °C with stirring, under an atmosphere of argon for 6.5 h. After cooling to rt, the solvent was evaporated *in vacuo* to give phthalimide **5** (31.7 g, quantitative) as a white powder that required no further purification.

TLC R_f = 0.45 (60% EtOAc in *n*-heptane, v/v).

IR (neat) v_{max}(neat) 3458, 2947, 1770, 1705, 1467, 1438, 1397, 1372, 1035 cm^{-1}.

^{1}**H NMR** (600 MHz, $CDCl_3$) δ 7.89-7.83 (m, 2H, Ar), 7.76-7.70 (m, 2H, Ar), 3.86 (t, *J* = 7, 6 Hz, 2H, CH_2OH), 3.62 (t, *J* = 6 Hz, 2H, NCH_2), 2.45 (br m, 1H, OH) 1.89 (quint, *J* = 6 Hz, 2H, NCH_2CH_2).

^{13}**C NMR** (151 MHz, $CDCl_3$) δ 168.9 (C=O), 134.1 (Ar), 132.0 (Ar), 123.4 (Ar), 59.0 (CH_2OH), 34.2 (NCH_2), 31.3 (NCH_2CH_2).

LRMS (ESI^+) *m/z*: $[M + H]^+$ calcd for $C_{11}H_{12}NO_3^+$ 206.1; found: 206.0.

The analytical data is in agreement with that previously reported [3].

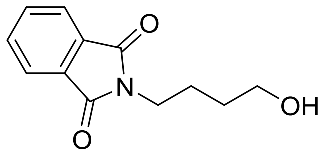

2-(4-Hydroxybutyl)isoindoline-1,3-dione (6)

4-Aminobutan-1-ol (1.78 g, 20 mmol) was added to a 100 ml round bottom flask fitted with a Dean–Stark receiver and suspended in dry toluene (60 ml). Phthalic anhydride (2.96 g, 20 mmol) was added and the mixture was heated to reflux with stirring for 3.5 h. The solvent was evaporated in vacuo and the residue purified by flash column chromatography (60% EtOAc in *n*-heptane, v/v) to give phthalimide **6** (3.32 g, 76%) as a white amorphous solid.

TLC R_f = 0.3 (60 % EtOAc in *n*-heptane, v/v).

HPLC t_R = 6.83 min.

IR (neat) v_{max} 3494, 2940, 1702, 1397, 1044 cm^{-1}.

LRMS (ESI^+) *m/z*: $[M + H]^+$ calcd for $C_{12}H_{13}NO_3^+$ 220.1; found: 220.1.

^{1}H NMR (600 MHz; $CDCl_3$): δ 7.87-7.82 (m, 2H, Ar), 7.74-7.69 (m, 2H, Ar), 3.75 (t, *J* = 7 Hz, 2H, CH_2N), 3.73-3.67 (m , 2H, CH_2OH), 1.83-1.74 (m, 2H, CH_2), 1.66-1.59 (m, 2H, CH_2), 1.36 (t, *J* = 5.5 Hz, 1H, OH).

^{13}C NMR (151 MHz, $CDCl_3$) δ 168.6 (C=O), 134.1 (Ar), 132.3 (Ar), 123.4 (Ar), 62.6 (CH_2OH), 37.9 (NCH_2), 29.9 (CH_2), 25.3 (CH_2).

The analytical data is in agreement with that previously reported [4].

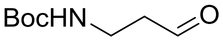

N-(tert-Butoxycarbonyl)-3-amino-propionaldehyde (7)

Alcohol **3** (1.0 g, 5.7 mmol) was dissolved in anhydrous DMSO (50 ml) under argon. Triethylamine (12 ml, 85.7 mmol) and sulfur trioxide pyridine complex (2.7 g, 17.1 mmol) were added and the mixture was stirred at rt for 4.5 h. The mixture was transferred to a separatory funnel with sulfate buffer (40 ml) and extracted with EtOAc (3 × 30 ml). The combined organic phases were washed with saturated aq. $NaHCO_3$ (90 ml), brine (90 ml), dried (Na_2SO_4) and concentrated in vacuo. Purification by flash column chromatography (40% EtOAc in *n*-heptane, v/v) gave aldehyde **7** (0.46 g, 47%) as a colourless oil.

TLC R_f = 0.25 (40% EtOAc in *n*-heptane, v/v).

^{1}H NMR (600 MHz, $CDCl_3$) δ 9.80 (br s, 1H, CHO), 4.89 (br s, 1H, NH), 3.42 (q, *J* = 6 Hz, 2H, NHCH_2), 2.70 (t, *J* = 6 Hz, 2H, CH_2CHO), 1.42 (s, 9H, $C(CH_3)_3$).

^{13}C NMR (151 MHz, $CDCl_3$) δ 201.4 (CHO), 149.8 (NC=O), 79.5 (*C*$(CH_3)_3$), 44.3 (*C*H_2CHO), 34.0 ($NHCH_2$), 28.4 (C(*C*$H_3)_3$).

The analytical data is in agreement with that previously reported [5].

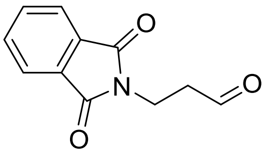

3-(1,3-Dioxoisoindolin-2-yl)propanal (9)

A 50 ml round bottom flask was charged with aq. $NaHCO_3$ (0.05 M, 10 ml), aq. K_2CO_3 (0.5 M, 10 ml) and $CHCl_3$ (5 ml). Alcohol **5** (16.02 g, 78.1 mmol) was dissolved in $CHCl_3$ (50 ml) and transferred to the reaction mixture followed by tetra-*n*-butylammonium chloride (4.34 g, 15.6 mmol), *N*-chlorosuccinimide (20.9 g, 156.1 mmol) and TEMPO (2.44 g, 15.6 mmol) and the reaction mixture was stirred vigorously at rt for 4.5 h. The phases were separated and the organic phase was washed with sulfate buffer (100 ml), $NaHCO_3$ (100 ml), and brine (100 ml), dried (Na_2SO_4), filtered and concentrated in vacuo. Purification by flash column chromatography (35% EtOAc in *n*-heptane, v/v) gave aldehyde **9** (6.54 g, 38%) as a white amorphous solid.

TLC R_f = 0.2 (30% EtOAc in *n*-heptane, v/v).

IR (neat) ν_{max} 1772, 1707, 1396, 1367, 1029 cm^{-1}.

^{1}H NMR (600 MHz, $CDCl_3$) δ 9.85 (t, *J* = 1.5 Hz, 1H, CHO), 7.90-7.85 (m, 2H, Ar), 7.77-7.73 (m, 2H, Ar), 4.07 (t, *J* = 7 Hz, 2H, NCH_2), 2.90 (td, *J* = 7, 1.5 Hz, 2H, CH_2CHO).

^{13}C NMR (151 MHz, $CDCl_3$) δ 199.5 (CHO), 168.2 (Pht-C=O), 134.3 (Ar), 132.1 (Ar), 123.5 (Ar), 42.5 (NCH_2), 31.9 (NCH_2CH_2).

The analytical data is in agreement with that previously reported [6].

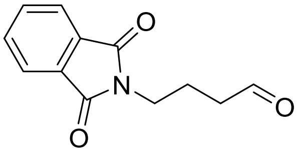

4-(1,3-Dioxoisoindolin-2-yl)butanal (10)

To a stirred solution of alcohol **6** (3.31 g, 15.1 mmol) in dry DMSO (100 ml), triethylamine (32 ml, 227 mmol) was added followed by sulfur trioxide pyridine complex (7.21 g, 45 mmol). After 1.5 hours the reaction was quenched with sulfate buffer (100 ml) and transferred to separating funnel with water (40 ml). The reaction mixture was extracted with EtOAc (3 × 75 ml) and the combined organic layers were washed with sat. $NaHCO_3$ (150 ml) and brine (150 ml), dried (Na_2SO_4), filtered and evaporated *in vacuo*. Purification by flash column chromatography (30% EtOAc in *n*-heptane, v/v) gave aldehyde **10** (2.74 g, 83%) as a white amorphous solid.

TLC R_f = 0.2 (30 % EtOAc in *n*-heptane, v/v).

HPLC t_R = 7.32 min.

IR (neat) v_{max} 1770, 1702, 1395, 1043 cm^{-1}.

LRMS (ESI^+) *m/z*: $[M + H]^+$ calcd for $C_{12}H_{11}NO_3^+$ 218.1; found: 218.1.

^{1}H NMR (600 MHz; $CDCl_3$) δ 9.78 (t, *J* = 1 Hz, 1H, CHO), 7.87-7.83 (m, 2H, Ar), 7.74-7.70 (m, 2H, Ar), 3.75 (t, *J* = 7 Hz, 2H, NCH_2), 2.54 (td, *J* = 7, 1 Hz, 2H, CH_2CHO), 2.02 (quint, *J* = 7 Hz, 2H, CH_2CH_2CHO).

^{13}C NMR (151 MHz, $CDCl_3$): δ 201.0 (CHO), 168.5 (Pht-C=O), 134.2 (Ar), 132.2 (Ar), 123.5 (Ar), 41.3 (CH_2CHO), 37.3 (NCH_2), 21.4 ($CH_2CH_2CH_2$).

The analytical data is in agreement with that previously reported [7].

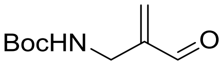

N-($tert$-Butoxycarbonyl)-3-amino-2-methylenepropanal (11)

Aldehyde **7** (0.43 g, 2.47 mmol) was dissolved in propan-2-ol (5 ml). 37% aq. formaldehyde (203 µl, 2.71 mmol), pyrrolidine (20 µl, 0.25 mmol) and propionic acid (18 µl, 0.25 mmol) were added and the reaction mixture was stirred at 45 °C for 26 h. The reaction mixture was transferred to a separating funnel with sulfate buffer (40 ml) and extracted with EtOAc (3 × 40 ml). The combined organic phases were washed with $NaHCO_3$ (90 ml), and brine (90 ml), dried (Na_2SO_4), filtered and concentrated in vacuo to give alkene **11** (0.37 g, 82%) as a white amorphous solid that required no further purification.

TLC R_f = 0.50 (40% EtOAc in *n*-heptane, v/v).

^{1}H NMR (600 MHz, $CDCl_3$) δ 9.61 (s, 1H, CHO), 6.44 (s, 1H, CH_aH_b), 6.12 (s, 1H, CH_aH_b), 4.95 (s, 1H, NH), 3.95 (d, J = 6.5 Hz, 2H, $NHCH_2$), 1.45 (s, 9H, $C(CH_3)_3$).

^{13}C NMR (151 MHz, $CDCl_3$) δ 194.0 (CHO), 155.7 (NC=O), 146.5 (C=CH_2), 134.7 (=CH_2), 79.7 ($C(CH_3)_3$), 38.8 ($NHCH_2$), 28.4 ($C(CH_3)_3$).

Compound **11** has been reported previously with no characterisation [8].

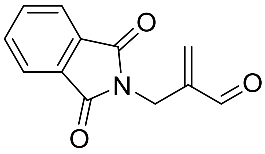

2-((1,3-Dioxoisoindolin-2-yl)methyl)acrylaldehyde (12)

Aldehyde **9** (6.54 g, 32.2 mmol) was dissolved in propan-2-ol (60 ml). 37% aq. formaldehyde (2.64 ml, 35.4 mmol), pyrrolidine (0.27 ml, 3.22 mmol) and propionic acid (0.24 ml, 3.22 mmol) were added and the reaction mixture was stirred at 45 °C for 24 h. The reaction mixture was transferred to a separating funnel with sulfate buffer (100 ml) and extracted with EtOAc (3 × 40 ml). The combined organic phases

were washed with $NaHCO_3$ (100 ml), and brine (100 ml), dried (Na_2SO_4), filtered and concentrated in vacuo to give alkene **12** (3.78 g, 55%) as a white amorphous powder that required no further purification.

TLC R_f = 0.4 (30% EtOAc in *n*-heptane, v/v).

IR (neat) v_{max} 2972, 1777, 1714, 1395, 1114, 960 cm^{-1}.

LRMS (ESI^+) *m/z*: $[M + H]^+$ calcd for $C_{12}H_{10}NO_3^+$ 216.1; found: 216.1.

^{1}H NMR (600 MHz, $CDCl_3$) δ 9.66 (s, 1H, CHO), 7.93-7.88 (m, 2H, Ar), 7.80-7.76 (m, 2H, Ar), 6.23 (t, *J* = 1.5 Hz, 1H, CH_aH_b), 6.18 (br s, 1H, CH_aH_b), 4.57 (d, *J* = 1.5 Hz, 2H, NCH_2).

^{13}C NMR (151 MHz, $CDCl_3$) δ 192.5 (CHO), 167.7 (Pht-C=O), 144.0 (C=CH_2), 134.2 (=CH_2), 133.9 (Ar), 132.0 (Ar), 123.5 (Ar), 35.6 (NCH_2).

The analytical data is in agreement with that previously reported [9].

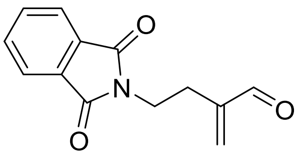

4-(1,3-Dioxoisoindolin-2-yl)-2-methylenebutanal (13)

Aldehyde **10** (66 mg, 0.30 mmol) was dissolved in propan-2-ol (4.3 ml) and warmed to 45 °C while stirring. 37% aq. formaldehyde (25 µl, 0.33 mmol), pyrrolidine (2.5 µl, 10 mol %) and propionic acid (2.3 µl, 10 mol %) were added. After 2 h the mixture was quenched with sulfate buffer (5 ml) and extracted with EtOAc (3 × 5 ml). The combined organic layers were washed with sat. $NaHCO_3$ (15 ml) and brine (15 ml), dried (Na_2SO_4), filtered and concentrated in vacuo to give alkene **13** (74 mg, quantitative) as a colourless oil that solidified upon standing.

TLC R_f = 0.25 (30 % EtOAc in *n*-heptane, v/v).

HPLC t_R = 7.90 min.

IR (neat) v_{max} 1704, 1686, 1394, 1106, 718 cm^{-1}.

LRMS (ESI^+) *m/z*: $[M + H]^+$ calcd for $C_{13}H_{11}NO_3^+$ 230.1; found: 230.0.

HRMS (MALDI-Orbitrap) *m/z*: $[M + H]^+$ calcd for $C_{13}H_{11}NO_3^+$ 230.0812; found: 230.0814.

^{1}H NMR (600 MHz, $CDCl_3$) δ 9.53 (s, 1H, CHO), 7.87-7.80 (m, 2H, Ar), 7.75-7.68 (m, 2H, Ar), 6.25 (s, 1H, C=CH_aH_b), 6.01 (s, 1H, C=CH_aH_b), 3.86 (t, *J* = 7 Hz, 2H, NCH_2), 2.67 (t, *J* = 7, 2H, NCH_2CH_2).

^{13}C NMR (151 MHz, $CDCl_3$) δ 193.9 (CHO), 168.3 (Pht-C=O), 146.9 (*C*=CH_2), 135.5 (C=*C*H_2), 134.1 (Ar), 132.1 (Ar), 123.4 (Ar), 36.5 (NCH_2), 27.7 (NCH_2CH_2).

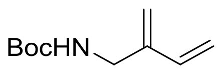

***N*-(*tert*-Butoxycarbonyl)-4-amino-3-methylenebutene (14)**

Methyltriphenylphosphonium iodide (41.8 g, 103 mmol) was suspended in anhydrous THF (350 ml) and cooled on a water-ice bath. Potassium *tert*-butoxide (11.6 g, 103 mmol) was added, to give a yellow slurry that was stirred for 30 min. Aldehyde **11** (17.4 g, 94 mmol) dissolved in anhydrous THF (150 ml) was added by cannula and the reaction was stirred vigorously at rt overnight (15 h). Half saturated aqueous NH_4Cl (50 ml) was added and the THF was removed in vacuo. The remaining reaction mixture was transferred to a separatory funnel with water (200 ml) and extracted with 20% EtOAc in hexanes (v/v) (1 × 200 ml and 3 × 100 ml). The combined organic phases were dried (Na_2SO_4), filtered and concentrated in vacuo to give a yellow gum. Purification by dry column vacuum chromatography [12] [id. 6 cm; 20 ml fractions; 10 × hexanes, 2–20% EtOAc in hexanes (v/v) – 2 % increments, 10 × 20% EtOAc in hexanes (v/v)] gave diene **14** (3.0 g, 17%) as a volatile colourless oil. The low yield is largely due to loss of material during removal of solvents in vacuo.

TLC R_f = 0.50 (30% EtOAc in *n*-heptane, v/v).

IR (neat) v_{max} 3257, 3056, 2978, 1702, 1437, 1180, 1118 cm^{-1}.

^{1}H NMR (600 MHz, $CDCl_3$) δ 6.40 (dd, *J* = 18, 11 Hz, 1H, C*H*=CH_2), 5.30 (d, *J* = 18 Hz, 1H, CH=CH_aH_b), 5.18-5.12 (m, 3H, CH=CH_aH_b and C=CH_2), 4.64 (s, 1H, NH), 3.97 (d, *J* = 6 Hz, 2H, NHCH_2), 1.47 (s, 9H, $C(CH_3)_3$).

^{13}C NMR (151 MHz, $CDCl_3$) δ 155.8 (C=O), 142.9 (*C*=CH_2), 136.7 (*C*H=CH_2), 116.2 (CH=*C*H_2), 114.3 (C=*C*H_2), 79.4 (*C*$(CH_3)_3$), 41.6 (NHCH_2), 28.4 (C(CH_3)$_3$).

Compound **14** has been reported previously with no characterisation [8].

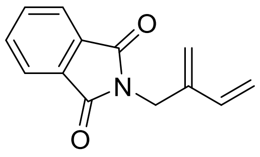

2-(2-Methylenebut-3-en-1-yl)isoindoline-1,3-dione (15)

Methyltriphenylphosphonium iodide (7.82 g, 19.3 mmol) was suspended in anhydrous THF (80 ml) and cooled on a water-ice bath. Potassium *tert*-butoxide (2.96 g, 26.4 mmol) was added, to give a pale yellow slurry that was stirred for 30 min. Aldehyde **12** (3.79 g, 17.6 mmol) was added and the reaction was stirred at rt for 24 h. The solvent was removed in vacuo to give a brown gum. Triphenylphosphine oxide was removed by the method of Lukin et al. [10] by dissolving the crude product in anhydrous toluene (100 ml) and adding $MgCl_2$ (3.68 g, 38.7 mmol). The mixture was diluted with *n*-heptane (100 ml) and stirred at 60 °C under an atmosphere of argon for 24 h. The reaction mixture was filtered, and the solids washed with toluene:*n*-heptane (1:1, 100 ml). The combined filtrates were evaporated in vacuo to give an oil. Purification by flash column chromatography (20% EtOAc in *n*-heptane, v/v) to give diene **15** as a white solid that required further purification. The product was dissolved in refluxing propan-2-ol (10 ml) and cooled slowly to give a white solid

that was isolated by filtration and washed with cold propan-2-ol to give diene **15** (0.35 g, 9%) as a white amorphous powder.

TLC R_f = 0.4 (20% EtOAc in *n*-heptane, v/v).

IR (neat) v_{max} 1772, 1710, 1397 cm^{-1}.

^{1}H NMR (600 MHz, $CDCl_3$) δ 7.89-7.85 (m, 2H, Ar), 7.75-7.70 (m, 2H, Ar), 6.43 (dd, *J* = 18, 11 Hz, 1H, C*H*=CH_2), 5.44 (d, *J* = 18 Hz, 1H, CH=C$H_a$$H_b$), 5.19 (s, 1H, C=C$H_a$$H_b$), 5.17 (d, *J* = 11 Hz, 1H, C=C$H_a$$H_b$), 5.05 (s, 1H, CH=C$H_a$$H_b$), 4.47 (s, 2H, NCH_2).

^{13}C NMR (151 MHz, $CDCl_3$) δ 168.3 (C=O), 139.8 (*C*=CH_2), 136.4 (*C*H=CH_2), 133.8 (Ar), 132.3 (Ar), 123.1 (Ar), 116.8 (C=*C*H_2), 114.7(C=*C*H_2), 38.7 (NCH_2).

LRMS (ESI^+) *m/z*: $[M + H]^+$ calcd for $C_{13}H_{12}NO_2^+$ 214.1; found: 214.1.

The analytical data is in agreement with that previously reported [11].

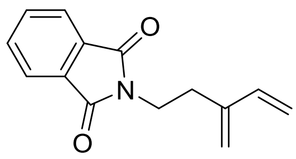

2-(3-Methylenepent-4-en-1-yl)isoindoline-1,3-dione (16)

Triphenylmethylphosphonium iodide (2.05 g, 5.1 mmol) was suspended in dry THF (35 ml). The mixture was cooled to 0 ºC (ice/water bath) under an atmosphere of argon. Potassium *tert*-butoxide (0.57 g, 5.1 mmol) was added in one portion to the solution, which turned yellow and was stirred for 30 min. Aldehyde **13** (0.78 g, 3.4 mmol) was dissolved in dry THF (3 ml) and added dropwise to the reaction flask and the slurry was stirred at ambient temperature over the weekend. The solution was quenched with sulfate buffer (50 ml) and extracted with EtOAc (3 × 50 ml). The combined organic layers were washed with sat. aq. $NaHCO_3$ (100 ml) and brine (100 ml), dried (Na_2SO_4), filtered and evaporated in vacuo to give an orange oil.

Purification by flash column chromatography (25 % EtOAc in *n*-heptane, v/v) gave diene **16** (0.41 g, 54%) as a white solid.

TLC R_f = 0.5 (25 % EtOAc in *n*-heptane, v/v).

HPLC t_R = 9.65 min.

IR (neat) v_{max} 1172, 1707, 1394, 1112 cm^{-1}.

LRMS (ESI^+) *m/z*: $[M + H]^+$ calcd for $C_{14}H_{14}NO_2^+$ 228.1; found: 228.1.

HRMS (MALDI-Orbitrap) *m/z*: $[M + H]^+$ calcd for $C_{14}H_{13}NO_2^+$ 228.1019; found: 228.1021.

^{1}H NMR (600 MHz, $CDCl_3$) δ 7.80-7.88 (m, 2H, Ar), 7.76-7.66 (m, 2H, Ar), 6.38 (dd, *J* = 18, 11 Hz, 1H, C*H*=CH_2), 5.43 (d, *J* = 18 Hz, 1H, *trans*-CH=CH_aH_b), 5.16 (d, *J* = 11 Hz, 1H, *cis*-CH=CH_aH_b), 5.08 (s, 1H, C=CH_aH_b), 5.07 (s, 1H, C=CH_aH_b), 3.88-3.82 (m, 2H, NCH_2), 2.65-2.58 (m, 2H, NCH_2CH_2).

^{13}C NMR (151 MHz, $CDCl_3$) δ 168.4 (C=O), 143.0 (*C*=CH_2), 138.0 (*C*=CH_2), 134.0 (Ar), 132.3 (Ar), 123.4 (Ar), 118.1 (CH_2=CH), 114.3 (CH_2=C), 37.3 (NCH_2), 30.6 (CH_2).

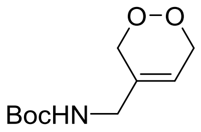

tert-Butyl [(3,6-dihydro-1,2-dioxin-4-yl)methyl]carbamate (17)

The photochemical reactor was charged with anhydrous CH_2Cl_2 (300 ml), diene **14** (3.0 g, 16.5 mmol) and rose bengal (0.30 mg, 2 mol%). The reaction mixture was water cooled and a gentle stream of O_2 was bubbled through the solution while irradiating with 3 × 100 W halogen lamps. Additional rose bengal (100 mg) was added when the colour was observed to fade (8, 16, and 24 h). After 32 h the solvent was evaporated in vacuo and the residue purified by dry column vacuum

chromatography [12] [id. 4 cm; 20 ml fract.; 8 × hexanes; 5–60% EtOAc in hexanes (v/v), 5% increments] to give endoperoxide **17** (0.91 g, 26%) as an amorphous solid.

TLC R_f = 0.35 (30% EtOAc in hexanes, v/v).

IR (neat) ν_{max} 3354, 2978, 1694, 1518, 1166 cm^{-1}.

HRMS (MALDI-orbitrap) *m/z*: $[M + Na]^+$ calcd for $C_{10}H_{17}NO_4Na$, 238.1050; found: 238.1053.

^{1}H NMR (600 MHz, $CDCl_3$) δ 5.84-5.82 (m, 1H, C=CH), 4.64 (br s, 1H, NH), 4.59-4.56 (m, 2H, CH_2O-O), 4.34-4.31 (m, 2H, CH_2O-O), 3.76 (d, *J* = 6 Hz, 2H, NCH_2), 1.44 (s, 9H, $(CH_3)_3$).

^{13}C NMR (151 MHz, $CDCl_3$) δ 156.0 (C=O), 133.9 (*C*=CH), 119.6 (C=*C*H), 80.0 (*C*$(CH_3)_3$), 71.0 (CH_2O-O), 69.9 (CH_2O-O), 42.3 (NCH_2), 28.5 (*C*$(CH_3)_3$).

Compound **17** has been reported previously with no characterisation [8].

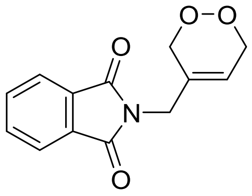

2-[(3,6-Dihydro-1,2-dioxin-4-yl)methyl]isoindoline-1,3-dione (18)

The photochemical reactor was charged with anhydrous CH_2Cl_2 (300 ml), diene **15** (0.48 g, 2.27 mmol) and tetraphenylporphyrin (TPP, 55 mg, 4 mol%). The reaction mixture was water cooled and a gentle stream of O_2 was bubbled through the solution while irradiating with 3 × 100 W halogen lamps. Additional TPP (30 mg) was added when the colour was observed to fade (10, 17, and 23 hr). After 28 h the solvent was evaporated *in vacuo* and the residue purified by flash column chromatography (30% EtOAc in *n*-heptane, v/v) to give endoperoxide **18** (0.28 g, 51%) as an amorphous solid.

TLC R_f = 0.3 (30% EtOAc in *n*-heptane, v/v).

IR (neat) ν_{max} 1710, 1395 cm^{-1}.

HRMS (MALDI-orbitrap) *m/z*: $[M + Na]^+$ calcd for $C_{13}H_{11}NO_4Na$, 268.0580; found: 268.0582.

^{1}H NMR (600 MHz, $CDCl_3$) δ 7.89-7.85 (m, 2H, Ar), 7.76-7.73 (m, 2H, Ar), 6.02 (br s, 1H, C=CH), 4.57 (br s, 4H, CH_2OOCH_2), 4.29 (s, 2H, NCH_2).

^{13}C NMR (151 MHz, $CDCl_3$) δ 168.0 (C=O), 134.2 (*C*=CH), 131.9 (Ar), 131.0 (Ar) 123.5 (Ar), 122.5 (C=*C*H), 71.0 (OCH_2), 69.7 (OCH_2), 38.9 (NCH_2).

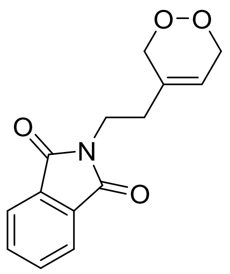

2-(2-(3,6-Dihydro-1,2-dioxin-4-yl)ethyl)isoindoline-1,3-dione (19)

By the same method as reported above anhydrous CH_2Cl_2 (300 ml), diene **16** (1.59 g, 7.01 mmol) and rose bengal bis(triethylammonium) salt (143 mg, 2 mol %) suspended in CH_2Cl_2 (1 ml) was irradiated for 40 h. Purification by flash column chromatography (30 % EtOAc in *n*-heptane, v/v) gave endoperoxide **19** (0.10 g, 6%) and recovered starting material **15** (1.24 g, 78%).

By the same method using TPP (0.14 g, 4 mol %) and diene **15** (1.3 g, 5.72 mmol) in CH_2Cl_2 (300 ml) after 35 h gave endoperoxide **19** (1.18 g, 80%) as tan powder and recovered starting material **15** (54 mg, 4%).

TLC R_f = 0.3 (30 % EtOAc in *n*-heptane, v/v).

HPLC t_R = 8.41 min.

IR (neat) ν_{max} 1171, 1704, 1396, 1362 cm^{-1}.

LRMS (ESI^+) *m/z*: $[M + H]^+$ calcd for $C_{14}H_{13}NO_3$, 260.1; found: 260.0.

HRMS (MALDI-orbitrap) *m/z*: $[M + Na]^+$ calcd for $C_{13}H_{13}NO_3Na$, 282.0737; found: 282.0738.

^{1}H NMR (600 MHz, $CDCl_3$) δ 7.90-7.80 (m, 2H, Ar), 7.76-7.68 (m, 2H, Ar), 5.80-5.69 (m, 1H, C=CH), 4.59-4.53 (m, 2H, CH_2O), 4.53-4.48 (m, 2H, CH_2O), 3.83 (t, *J* = 7 Hz, 2H, NCH_2), 2.45 (t, *J* = 7.5 Hz, 2H, NCH_2CH_2).

^{13}C NMR (151 MHz, $CDCl_3$) δ 168.3 (C=O), 134.2 (Ar), 132.4 (Ar), 132.2 (C=CH), 123.5 (Ar), 120.2 (CH=C), 72.1 (CO-O), 70.0 (CO-O), 36.2 (CH_2), 31.4 (CH_2).

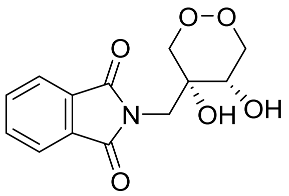

(±)-2-[((4*R*,5*S*)-4,5-Dihydroxy-1,2-dioxan-4-yl)methyl]isoindoline-1,3-dione (20)

Endoperoxide **18** (150 mg, 0.61 mmol) was dissolved in *t*BuOH (2 ml), H_2O (2 ml), and CH_3CN (1 ml). K_2OsO_4 (1.1 mg, 3.1 µmol), NMO (79 mg, 0.67 mmol) and citric acid (0.24 g, 1.22 mmol) were added and the mixture was stirred at rt After 23 h additional NMO (79 mg, 0.67 mmol) was added and the reaction mixture was stirred for 4 h. The reaction mixture was diluted with H_2O (10 ml) and extracted with EtOAc (4 × 15 ml). The combined organic phases were washed with brine (40 ml), dried (Na_2SO_4), filtered and concentrated in vacuo to give diol **20** (0.15 g, 89%) as a solid that required no further purification.

TLC R_f = 0.3 (50% EtOAc in *n*-heptane, v/v).

IR (neat) ν_{max} 3451, 1708, 1395, 1049, 1009 cm^{-1}.

HRMS (MALDI-orbitrap) *m/z*: $[M + H]^+$ calcd for $C_{13}H_{14}NO_6$, 280.0821; found: 280.0820.

^{1}H NMR (600 MHz, $CDCl_3$) δ 7.91-7.89 (m, 2H, Ar), 7.80-7.78 (m, 2H, Ar), 4.26-4.23 (m, 2H, CHOHC*H_2*), 4.18 (d, *J* = 13.5 Hz, 1H, COCH_aH$_b$), 4.14-4.11 (m, 1H, COCH$_a$$H_b$), 4.01 (d, *J* = 15 Hz, 1H, NCH_aH$_b$), 3.84 (d, *J* = 15 Hz, 1H, NCH$_a$$H_b$), 3.75 (br s, 1H, C*H*OH), 3.36 (br s, 1H, COH), 3.05 (br s, 1H, CHO*H*).

^{13}C NMR (151 MHz, $CDCl_3$) δ 169.3 (C=O), 134.9 (Ar), 131.7 (Ar), 124.0 (Ar), 76.3 (COH), 72.5 (CHOH*C*H_2), 70.4 (*C*CH_2O), 66.0 (CHOH), 41.9 (NCH_2).

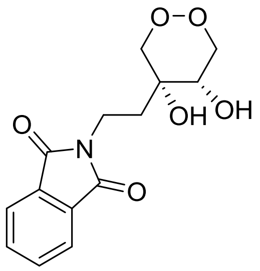

(±)-2-[2-((4*R*,5*S*)-4,5-Dihydroxy-1,2-dioxan-4-yl)ethyl]isoindoline-1,3-dione (21)

Endoperoxide **19** (96 mg, 0.37 mmol) was dissolved in *t*BuOH (2 ml), H_2O (1 ml) and CH_3CN (1 ml). Citric acid (0.15 g, 0.74 mmol), NMO (49 mg, 0.41 mmol) and K_2OsO_4 (3 mg, 8 µmol, dissolved in H_2O, 1 ml) were added. After stirring at ambient temperature overnight, additional K_2OsO_4 (4 mg, 11 µmol) and NMO (45 mg, 0.38 mmol) were added. After 1.5 h the reaction was diluted with H_2O (5 ml) and extracted with EtOAc (4 × 10 ml). The combined organic layers were washed with brine (20 ml), dried (Na_2SO_4), filtered and evaporated in vacuo to give diol **21** (0.10 g, 95%) as white amorphous solid that required no further purification.

TLC R_f = 0.1 (40 % EtOAc in *n*-heptane, v/v).

HPLC t_R = 6.63 min.

IR (neat) ν_{max} 3469, 2927, 1703, 1403 cm^{-1}.

LRMS (ESI^+) *m/z*: $[M + H]^+$ calcd for $C_{14}H_{15}NO_6$, 293.9; found: 294.1.

HRMS (MALDI-orbitrap) *m/z*: $[M + Na]^+$ calcd for $C_{14}H_{15}NO_6Na$, 316.0792; found: 316.0790.

^{1}H NMR (600 MHz, $CDCl_3$) δ 7.88-7.82 (m, 2H, Ar), 7.75-7.70 (m, 2H, Ar), 4.31-4.07 (m, 4H, CH_2O), 4.01-3.81 (m, 2H, NCH_2), 3.79-3.69 (m, 1H, C*H*OH), 2.75 (s, 1H, COH), 2.43 (d, *J* = 9 Hz, 1H, CHO*H*), 2.21 (ddd, *J* = 14.5, 8, 6.5 Hz, 1H, $NCH_2C\mathit{H}_aH_b$), 1.89 (ddd, *J* = 14.5, 8, 6.5, 1H, $NCH_2CH_a\mathit{H}_b$).

^{13}C NMR (151 MHz, $CDCl_3$) δ 168.6 (C=O), 134.2 (Ar), 132.2 (Ar), 123.5 (Ar), 77.8 (CO-O), 73.6 (CO-O), 69.1 (COH), 68.6 (COH), 33.2 (CH_2), 32.8 (CH_2).

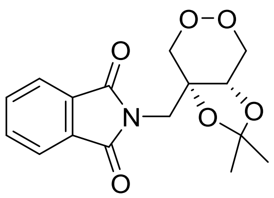

2-(((3a*R*,7a*S*)-2,2-Dimethyldihydro-[1,3]dioxolo[4,5-d][1,2]dioxin-3a(4*H*)-yl)methyl)isoindoline-1,3-dione (22)

Diol **20** (150 mg, 0.54 mmol) was dissolved in CH_2Cl_2 (5 ml). 2,2-Dimethoxypropane (198 µl, 1.61 mmol) was added, followed by *p*-toluenesulfonic acid (10 mg, 0.05 mmol) and the mixture was stirred at rt for 2 h under an atmosphere of argon. The mixture was diluted with CH_2Cl_2 (20 ml) and washed with $NaHCO_3$ (20 ml), dried (Na_2SO_4), filtered and concentrated in vacuo to give acetonide **22** (152 mg, 89%) that required no further purification.

TLC R_f = 0.35 (30 % EtOAc in *n*-heptane, v/v).

IR (neat) ν_{max} 2986, 2929, 1775, 1716, 1395, 1215, 1093, 1046 cm^{-1}.

HRMS (MALDI-orbitrap) *m/z*: $[M + H]^+$ calcd for $C_{16}H_{18}NO_6$, 320.1128; found: 320.1128.

^{1}H NMR (600 MHz, $CDCl_3$) δ 7.91-7.87 (m, 2H, Ar), 7.78-7.74 (m, 2H, Ar), 4.65 (dd, J = 14, 2 Hz, 1H, $CHCH_aH_bO$), 4.40 (ddd, J = 14, 2.5, 1.5 Hz, 1H, $CHCH_aH_bO$), 4.33 (d, J = 13 Hz, 1H, CCH_aH_bO), 4.13-4.08 (m, 2H, $COCH_aH_b$, NCH_aH_b), 4.04 (br s, 1H, C*H*OH), 3.96 (d, J = 14.5 Hz, 1H, NCH_aH_b), 1.52 (s, 3H, CH_3), 1.22 (s, 3H, CH_3).

^{13}C NMR (151 MHz, $CDCl_3$) δ 168.6 (C=O), 134.5 (Ar), 131.9 (Ar), 123.8 (Ar), 109.6 ($C(CH_3)_2$), 76.5 (CCH_2O), 75.8 (NCH_2C), 72.7 (*C*HOH), 71.5 ($CHCH_2O$), 41.1 (NCH_2), 28.1 (CH_3), 26.6 (CH_3).

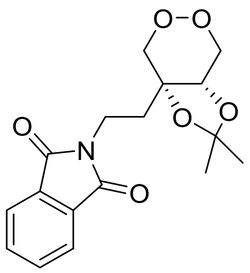

(±)-2-(2-((3a*R*,7a*S*)-2,2-Dimethyldihydro-[1,3]dioxolo[4,5-d][1,2]dioxin-3,a(4*H*)-yl)ethyl)isoindoline-1,3-dione (23)

Diol **21** (186 mg, 0.63 mmol) was dissolved in anhydrous CH_2Cl_2 (6 ml) and 2,2-dimethoxypropane (0.23 ml, 1.90 mmol) was added followed by *p*-toluenesulfonic acid (12 mg, 10 mol%). The reaction was stirred for 1 h under argon, diluted with CH_2Cl_2 (20 ml), washed with saturated aq. $NaHCO_3$ (20 ml), dried (Na_2SO_4), filtered and evaporated *in vacuo*. Purification by flash column chromatography (30% EtOAc in *n*-heptane, v/v) gave acetonide **23** (155 mg, 74%) as white amorphous solid.

TLC R_f = 0.3 (30 % EtOAc in *n*-heptane, v/v).

HPLC t_R = 8.85 min.

IR (neat) v_{max} = 1708, 1374, 1008 cm^{-1}.

LRMS (ESI^+) *m/z*: $[M + H]^+$ calcd for $C_{17}H_{19}NO_6$, 334.1; found: 334.1.

HRMS (MALDI-orbitrap) *m/z*: $[M + Na]^+$ calcd for $C_{17}H_{19}NO_6Na$, 356.1104; found: 356.1106.

^{1}H NMR (600 MHz, $CDCl_3$) δ 7.88-7.82 (m, 2H, Ar), 7.75-7.69 (m, 2H, Ar), 4.51 (dd, *J* = 14, 2 Hz, 1H, $CHC\mathit{H}_aH_bO$-O), 4.41 (ddd, *J* = 14, 2.5, 1.5 Hz, 1H, $CHCH_a\mathit{H}_bO$-O), 4.27 (d, *J* = 13 Hz, 1H, $CC\mathit{H}_aH_bO$-O), 4.11 (ddd, *J* = 13, 2.5, 1 Hz, 1H, $CCH_a\mathit{H}_bO$-O), 3.97-3.90 (m, 2H, $NC\mathit{H}_aH_b$ and OCH), 3.84 (ddd, *J* = 14, 9.5, 4.5 Hz, 1H, $NCH_a\mathit{H}_b$), 2.20-2.14 (m, 1H, $NCH_2C\mathit{H}_aH_b$), 2.11 (ddd, *J* = 14, 9, 4.5 Hz, 1H, $NCH_2CH_a\mathit{H}_b$), 1.44 (s, 3H, CH_3), 1.31 (s, 3H, CH_3).

^{13}C NMR (151 MHz, $CDCl_3$) δ 168.4 (C=O), 134.1 (Ar), 132.4 (Ar), 123.3 (Ar), 109.6 ($\mathit{C}(CH_3)_2$), 76.2 ($\mathit{C}CH_2O$), 75.2 ($CH_2\mathit{C}O$), 74.9 (CHO), 71.6 ($CH\mathit{C}H_2O$), 33.2 (NCH_2), 32.7 ($NCH_2\mathit{C}H_2$), 28.2 (CH_3), 27.1 (CH_3).

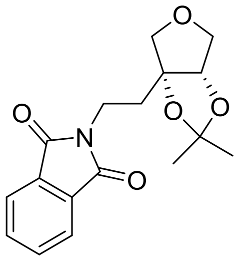

(±)-2-(2-((3*aR*,6*aS*)-2,2-Dimethyldihydrofuro[3,4-d][1,3]dioxol-3a(4*H*)-yl)ethyl)isoindoline-1,3-dione (24)

Endoperoxide **23** (50 mg, 0.15 mmol) was dissolved in anhydrous CH_2Cl_2 (1.5 ml), triphenylphosphine (60 mg, 0.23 mmol) was added and the reaction was refluxed with stirring under an atmosphere of argon. After 20 h the reaction was concentrated *in vacuo* to give a colourless oil. Purification by flash column chromatography (30 % EtOAc in *n*-heptane, v/v) gave tetrahydrofuran **24** (36 mg, 75%) as a colourless oil, that solidified to give a white amorphous solid.

TLC R_f = 0.2 (30 % EtOAc in *n*-heptane, v/v).

HPLC t_R = 8.32 min.

IR (neat) ν_{max} 1708, 1399, 1372, 1100 cm^{-1}.

HRMS (MALDI-Orbitrap) *m/z*: $[M + H]^+$ calcd for $C_{17}H_{20}NO_5^+$ 318.1336; found: 318.1335.

^{1}H NMR (600 MHz, $CDCl_3$) δ 7.88-7.82 (m, 2H, Ar), 7.74-7.69 (m, 2H, Ar), 4.47 (d, J = 3.5 Hz, 1H, $CHCH_2$), 4.06 (d, J = 10 Hz, 1H, $C_qCH_aH_b$), 4.01 (d, J = 11 Hz, 1H, $CHCH_aH_b$), 3.92 (ddd, J = 14, 9.5, 6.5 Hz, 1H, NCH_aH_b), 3.78 (ddd, J = 14, 10, 5 Hz, 1H, NCH_aH_b), 3.56 (dd, J = 11, 3.5 Hz, 1H, $CHCH_aH_b$), 3.47 (d, J = 10 Hz, 1H, $C_qCH_aH_b$), 2.25 (ddd, J = 14, 10, 6.5 Hz, 1H, $NCH_2CH_aH_b$), 2.05 (ddd, J = 14, 9.5, 5 Hz, 1H, $NCH_2CH_aH_b$), 1.43 (s, 3H, CH_3), 1.36 (s, 3H, CH_3).

^{13}C NMR (151 MHz, $CDCl_3$) δ 168.4 (C=O), 134.1 (Ar), 132.4 (Ar), 123.4 (Ar), 113.1 ($C(CH_3)_2$), 91.0 ($C_qOC(CH_3)_2$), 86.2 ($CHOC(CH_3)_2$), 77.8 (CCH_2O), 74.1 ($CHCH_2O$), 34.5 (NCH_2), 34.4 (NCH_2CH_2), 27.7 (CH_3), 27.6 (CH_3).

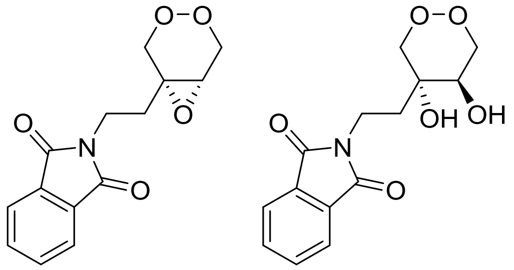

(±)-2-(2-((1*R*,6*S*)-3,4,7-Trioxabicyclo[4,1,0]heptan-1-yl)ethyl)-isoindoline-1,3-dione (25), and

(±)-2-(2((4*R*,5*R*)-4,5-dihydroxy-1,2-dioxan-4-yl)ethyl)isoindoline-1,3-dione (26)

To a solution of endoperoxide **19** (100 mg, 0.39 mmol) in anhydrous CH_2Cl_2 (2 ml) *m*CPBA (77%, 0.26 g, 1.16 mmol) was added in one portion and the reaction was stirred for 1 week. The reaction was diluted with CH_2Cl_2 (10 ml) and washed with saturated aq. sodium thiosulfate (2 × 4 ml), saturated aq. $NaHCO_3$ (4 ml) and brine (4 ml). The organic layers were dried (Na_2SO_4), filtered and evaporated *in vacuo*.

Purification by flash column chromatography (60% EtOAc in *n*-heptane, v/v) gave epoxide **25** (44 mg, 41%) and diol **26** (24 mg, 22%) as a white amorphous solids.

Analytical data for epoxide **25**:

TLC R_f = 0.6 (60 % EtOAc in *n*-heptane, v/v).

HPLC t_R = 7.69 min.

IR (neat) v_{max} 2912, 1699, 1403 cm^{-1}.

^{1}H NMR (600 MHz, $CDCl_3$) δ 7.89-7.83 (m, 2H, Ar), 7.76-7.70 (m, 2H, Ar), 4.43 (d, *J* = 13.5 Hz, 1H, CH_aH_bO-O), 4.39 (d, *J* = 13.5 Hz, 1H, CH_aH_bO-O), 4.36-4.29 (m, 2H, CH_aH_bO-O and $CHaH_bO$-O), 3.91-3.75 (m, 2H, NCH_2), 3.32 (d, *J* = 3 Hz, 1H, COCH), 2.25- 2.13 (m, 1H, $NCH_2CH_aH_b$), 2.08 (dt, *J* = 14.5, 7.5 Hz, 1H, $NCH_2CH_aH_b$).

^{13}C NMR (151 MHz, $CDCl_3$) δ 168.3 (C=O), 134.3 (Ar), 132.2 (Ar), 123.6 (Ar), 72.3 (CO-O), 70.0 (CO-O), 55.9 (epoxide-CO), 54.0 (epoxide-CO), 33.5 (CH_2), 31.4 (CH_2).

LRMS (ESI^+) *m/z*: $[M+H]^+$ calcd for $C_{14}H_{13}NO_5^+$ 276.1; found: 276.1.

HRMS (MALDI-Orbitrap) *m/z*: $[M+H]^+$ calcd for $C_{14}H_{14}NO_5^+$ 276.0866; found: 276.0862.

Analytical data for diol **26**:

TLC R_f = 0.25 (60 % EtOAc in *n*-heptane, v/v).

HPLC t_R = 6.27 min.

IR (neat) v_{max} 3567, 3417, 3020, 1702, 1214 cm^{-1}.

LRMS (ESI^+) *m/z*: $[M + H]^+$ calcd for $C_{14}H_{15}NO_6^+$ 294.1; found: 293.8.

HRMS (MALDI-Orbitrap) *m/z*: $[M + Na]^+$ calcd for $C_{14}H_{15}NO_6Na^+$ 316.0791; found: 316.0790.

^{1}H NMR (600 MHz, $CDCl_3$) δ 7.90-7.81 (m, 2H, Ar), 7.77-7.68 (m, 2H, Ar), 4.76 (d, *J* = 13.5 Hz, 1H, $CHCH_a$HbO-O), 4.48 (d, *J* = 13 Hz, 1H, CCH_aH_bO-O), 4.05 (dt, *J* = 13.5, 3 Hz, 1H, $CHCH_aH_bOO$), 3.97 (dt, *J* = 15, 7.5 Hz, 1H, NCH_aH_b), 3.86 (ddd, *J* = 14, 8, 5.5 Hz, 1H, NCH_aH_b), 3.80-3.74 (m, 2H, OHC*H* and CCH_aH_bO-O), 2.60-2.46

(m, 2H, 2 × OH), 2.11-2.01 (m, 1H, $NCH_2CH_aH_b$), 1.86 (dt, J = 15, 8 Hz, 1H, $NCH_2CH_aH_b$).

^{13}C NMR (151 MHz, $CDCl_3$) δ 168.7 (C=O), 134.2 (Ar), 132.2 (Ar), 123.5 (Ar), 77.6 (CO-O), 74.9 (CO-O), 69.2 (COH), 67.6 (COH), 32.0 (CH_2), 31.4 (CH_2).

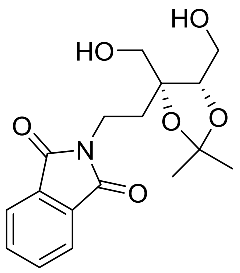

(±)-2-(2-((4*R*,5*S*)-4,5-bis(Hydroxymethyl)2-2-dimethyl-1,3-dioxolan-4-yl)ethyl)isoindoline-1,3-dione (28)

To a solution of endoperoxide **23** (150 mg, 0.45 mmol) in methanol (4 ml) was added palladium on carbon (15 mg, 10% w/w) and the mixture was stirred for 4 h under an atmosphere of hydrogen. The suspension was filtered through a pad of Celite, washed with methanol (ca. 25 ml) and evaporated *in vacuo* to give a colourless oil. Purification by flash column chromatography (5% methanol in CH_2Cl_2, v/v) gave diol **28** (14 mg, 10%) as a white amorphous solid.

TLC R_f = 0.25 (5% MeOH in CH_2Cl_2, v/v).

HPLC t_R = 7.20 min.

IR (neat) v_{max} 3462, 2986, 2936, 1707, 1052 cm^{-1}.

LRMS (ESI^+) *m/z*: $[M + H]^+$ calcd for $C_{17}H_{21}NO_6^+$ 336.2; found: 335.9.

HRMS (MALDI-Orbitrap) *m/z*: $[M + H]^+$ calcd for $C_{17}H_{22}NO_6^+$ 336.1441; found: 336.1441.

^{1}H NMR (600 MHz, $CDCl_3$) δ 7.86-7.80 (m, 2H, Ar), 7.74-7.68 (m, 2H, Ar), 4.02-3.95 (m, 2H, NCH_aH_b and OCH), 3.95-3.86 (m, 2H, CH_2OH), 3.81 (ddd, J = 14, 10, 4 Hz,

1H, NCH_aH_b), 3.74-3.64 (m, 2H, CH_2OH), 3.00 (t, *J* = 6.5 Hz, 1H, OH), 2.81 (t, *J* = 6.5 Hz, 1H, OH), 2.16 (ddd, *J* = 14, 9, 4 Hz, 1H, $NCH_2CH_aH_b$), 2.03 (ddd, *J* = 14, 10, 7.5 Hz, 1H, $NCH_2CH_aH_b$), 1.35 (s, 3H, CH_3), 1.32 (s, 3H, CH_3).

^{13}C NMR (151 MHz, $CDCl_3$) δ 168.6 (C=O), 134.1 (Ar), 132.3 (Ar), 123.4 (Ar), 108.4 ($C(CH_3)_2$), 82.7 ($COC(CH_3)_2$), 82.0 ($COC(CH_3)_2$), 62.2 (CH_2OH), 60.5 (CH_2OH), 33.6 (CH_2), 33.5 (CH_2), 28.5 (CH_3), 26.6 (CH_3).

NMR Spectra
4-(1,3-Dioxoisoindolin-2-yl)-2-methylenebutanal (13)

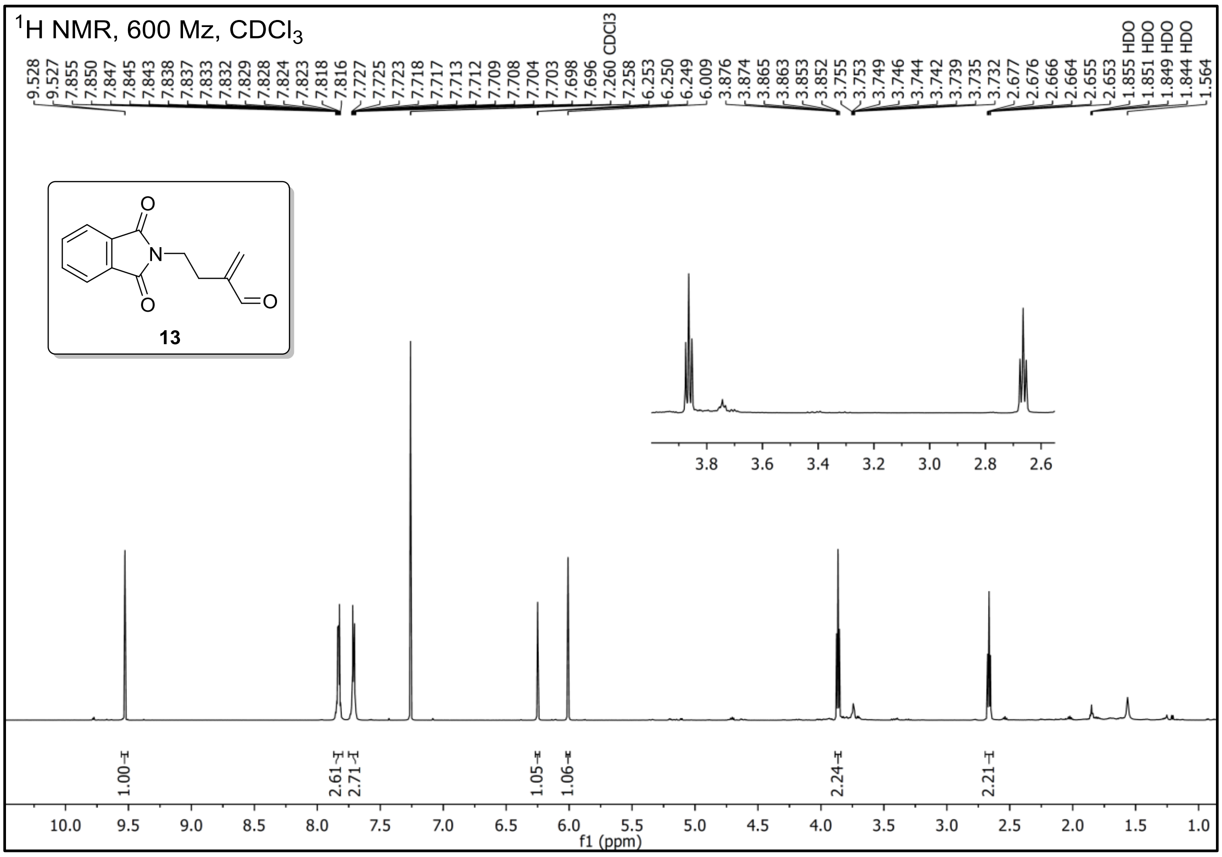

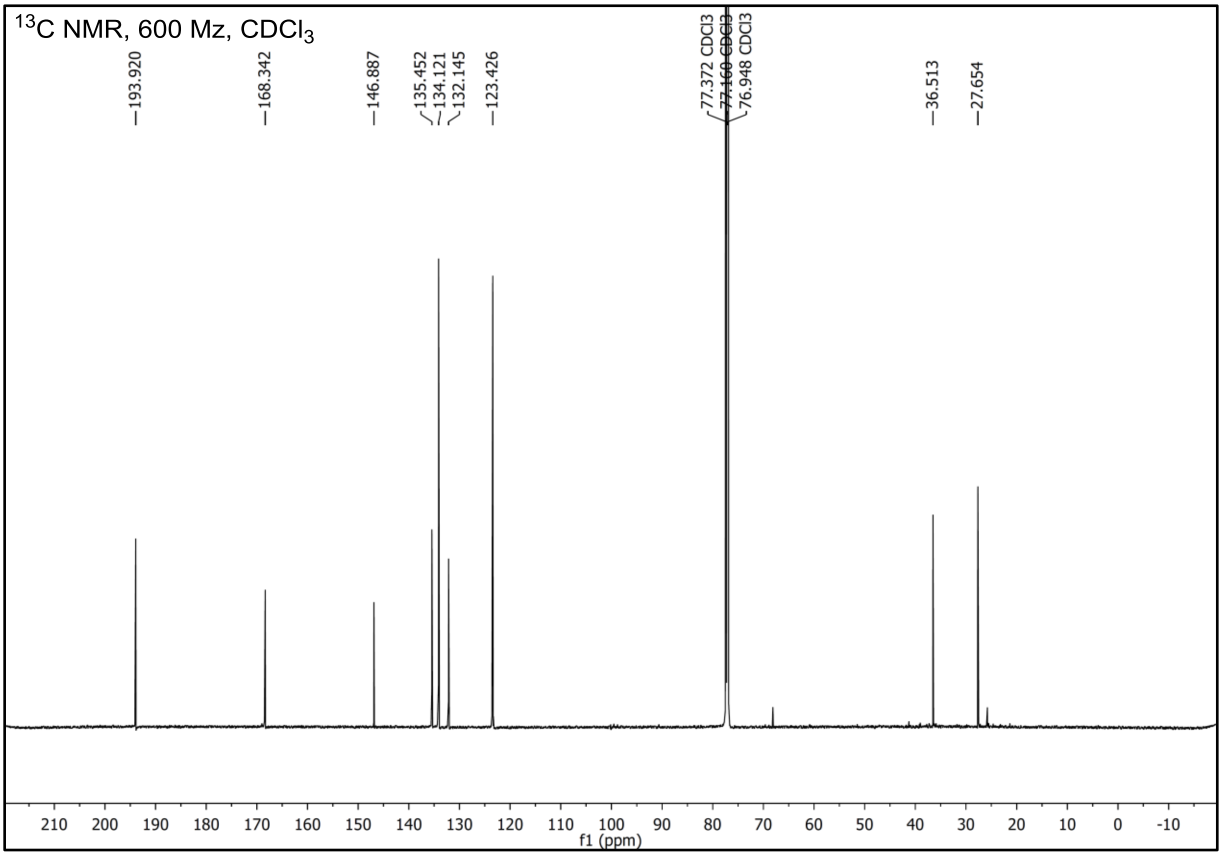

2-(3-Methylenepent-4-en-1-yl)isoindoline-1,3-dione (16)

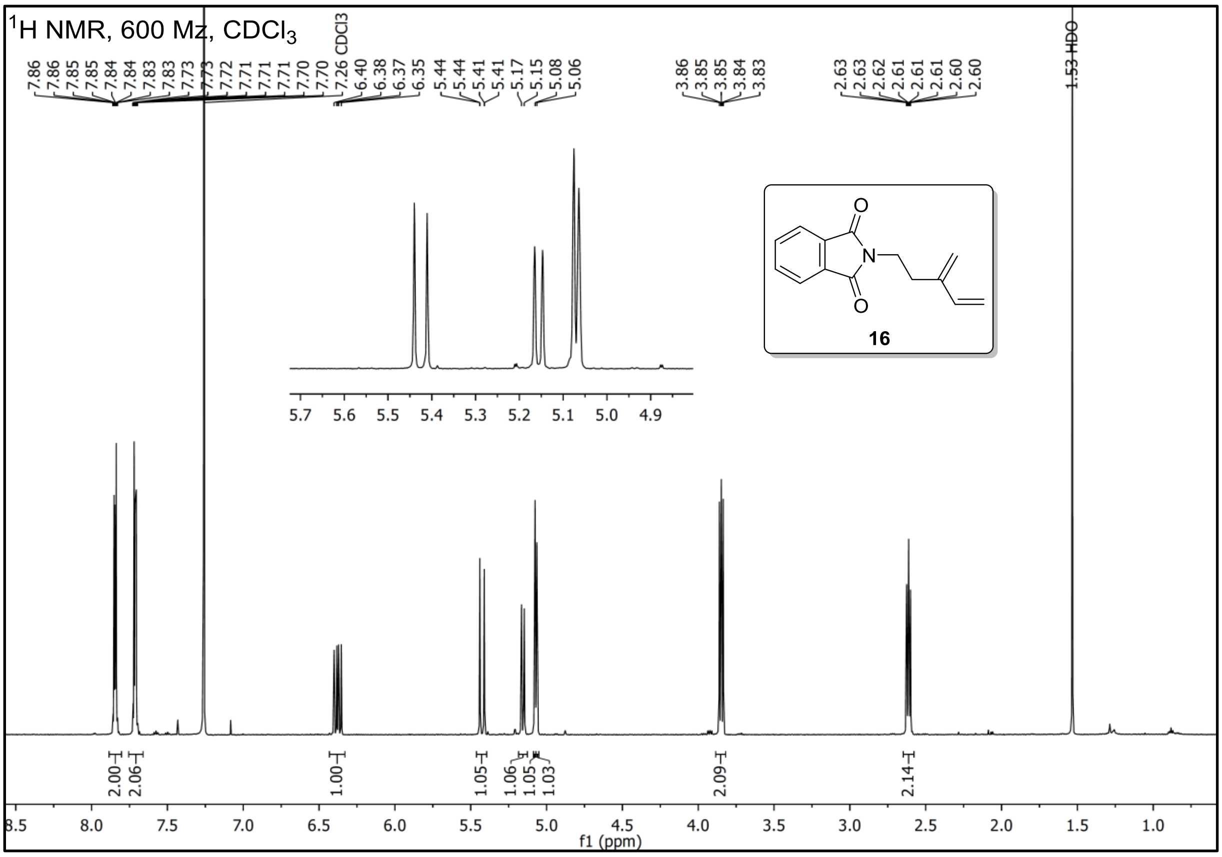

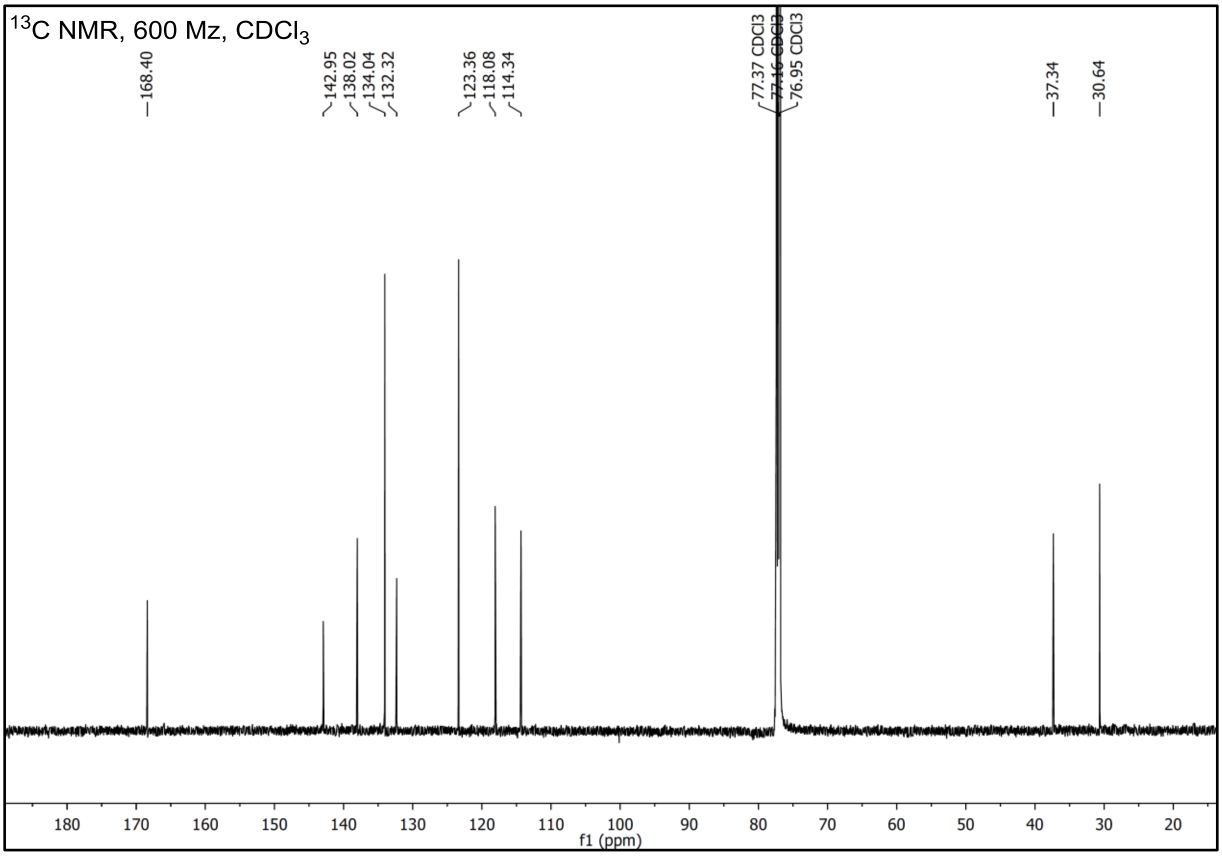

2-[(3,6-Dihydro-1,2-dioxin-4-yl)methyl]isoindoline-1,3-dione (18)

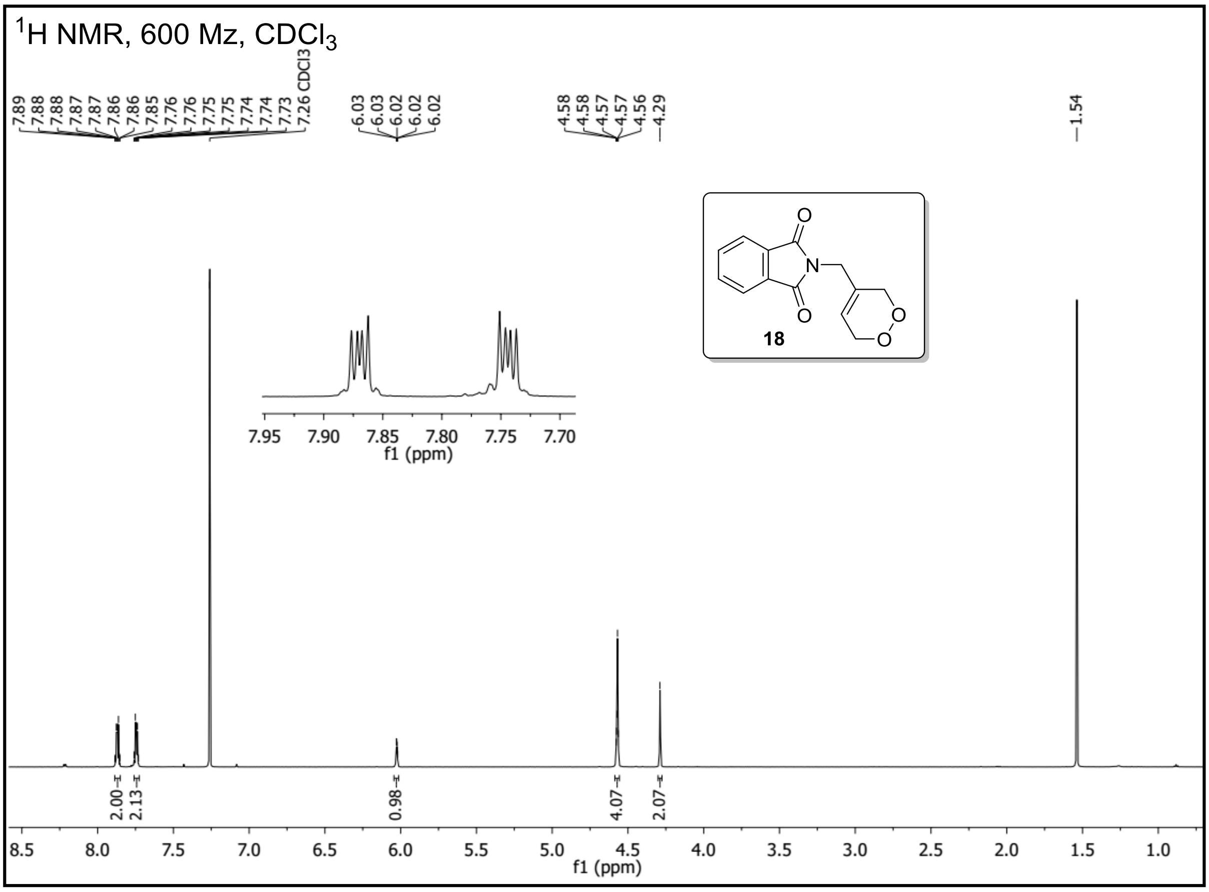

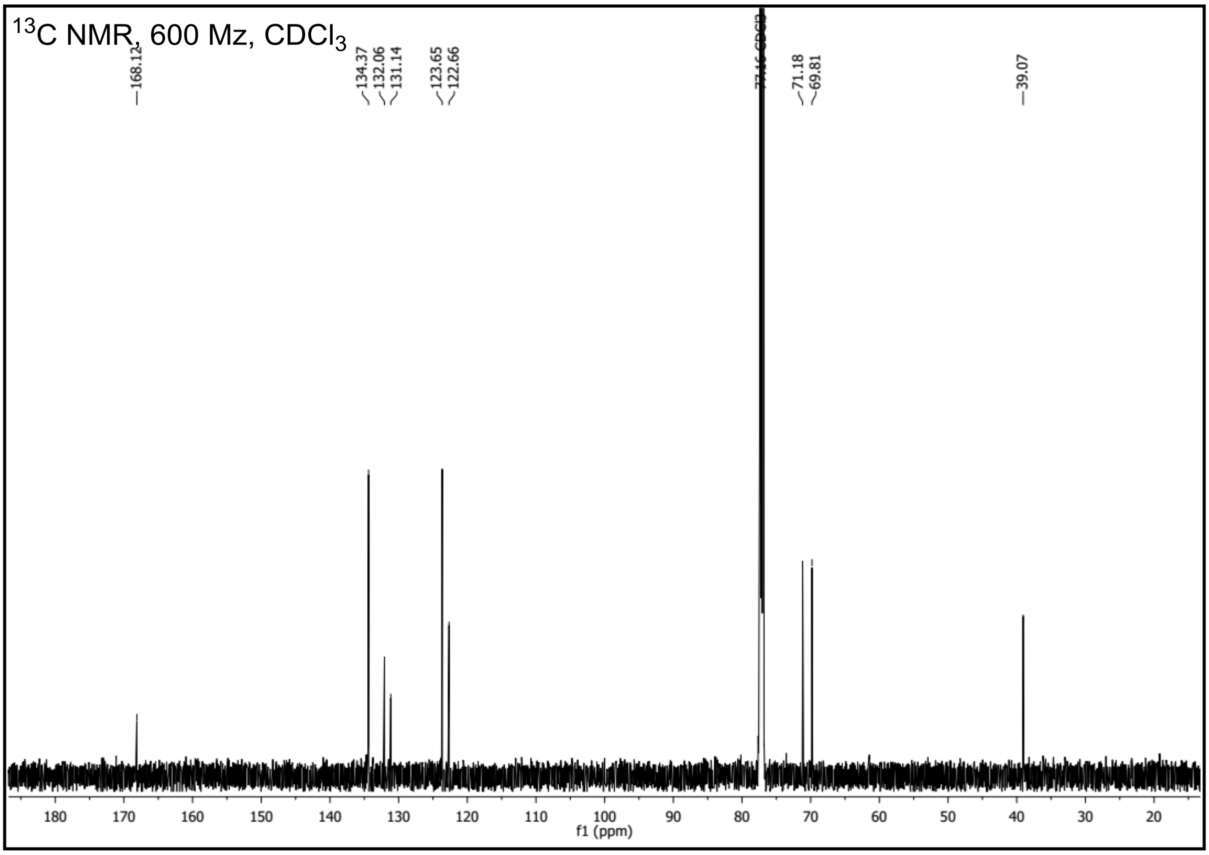

2-(2-(3,6-Dihydro-1,2-dioxin-4-yl)ethyl)isoindoline-1,3-dione) (19)

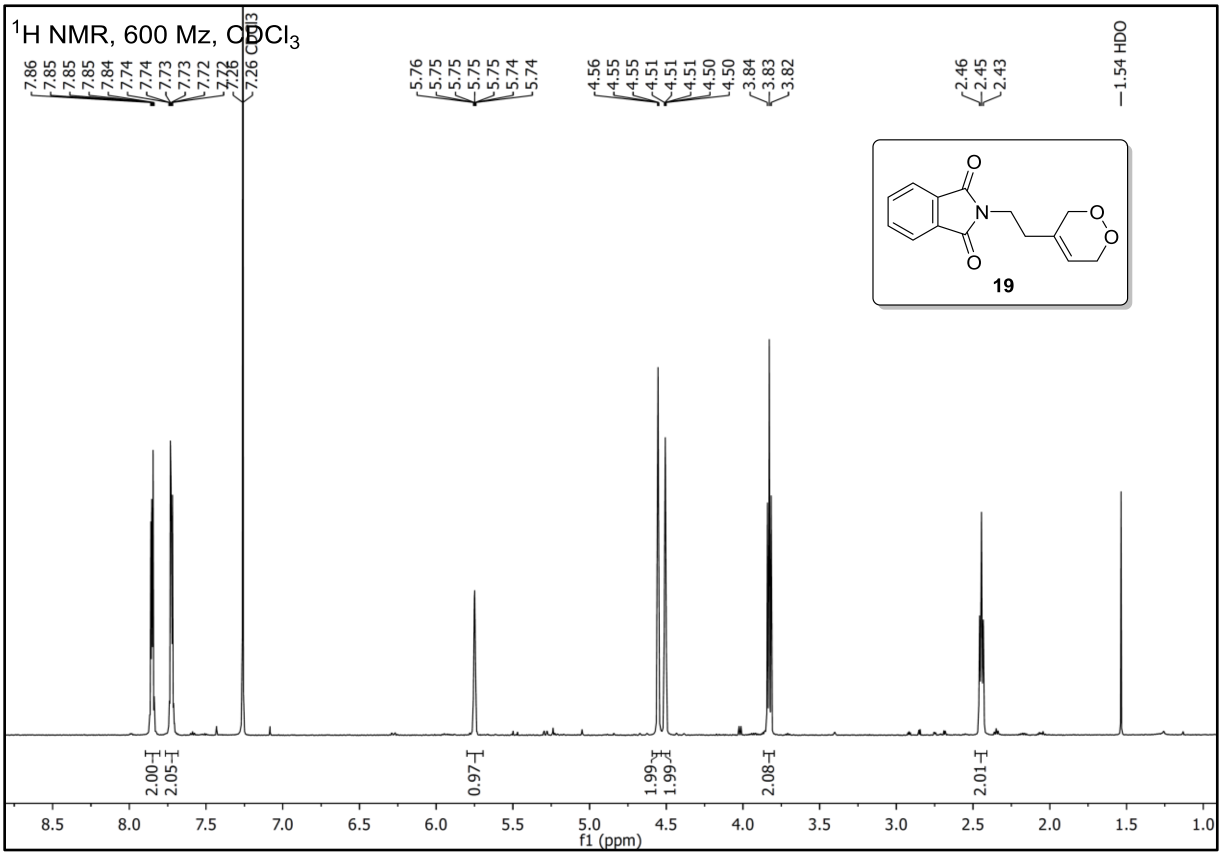

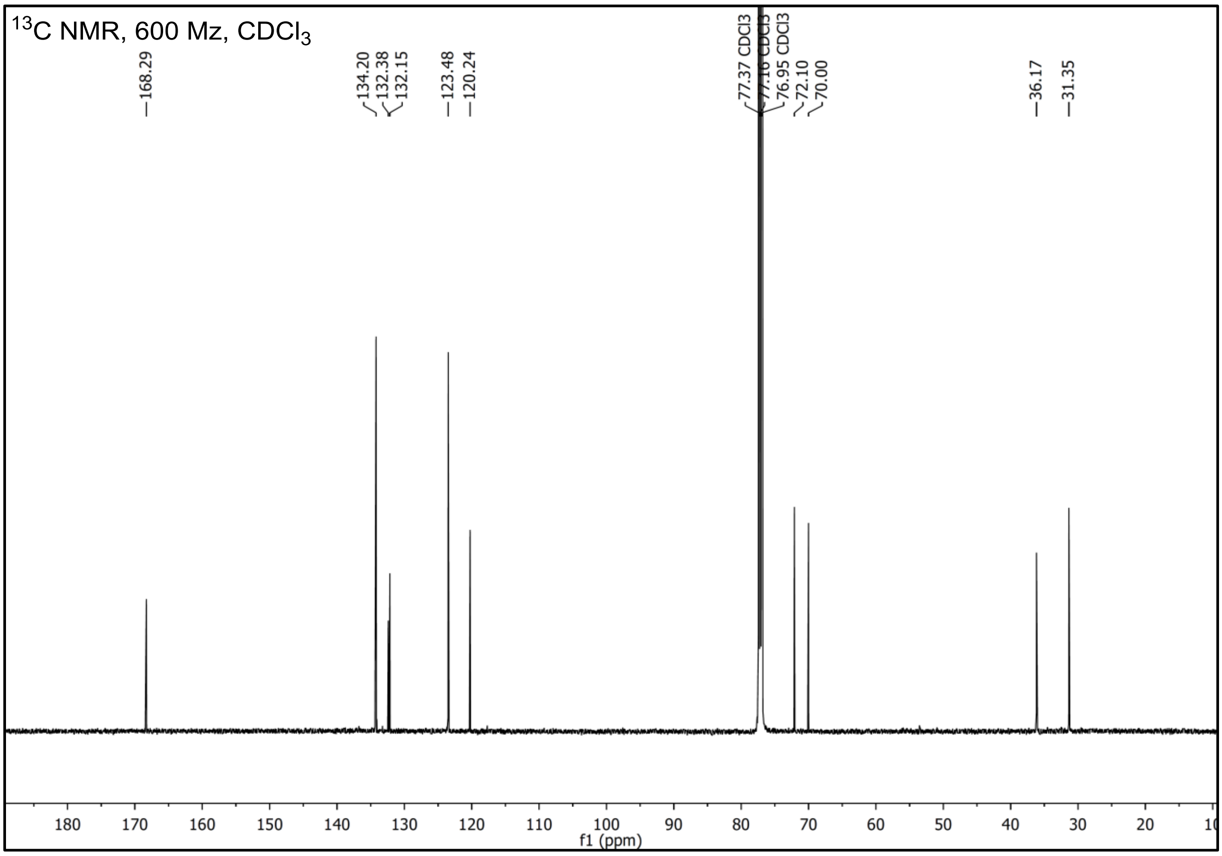

(±)-2-[((4*R*,5*S*)-4,5-Dihydroxy-1,2-dioxan-4-yl)methyl]isoindoline-1,3-dione (20)

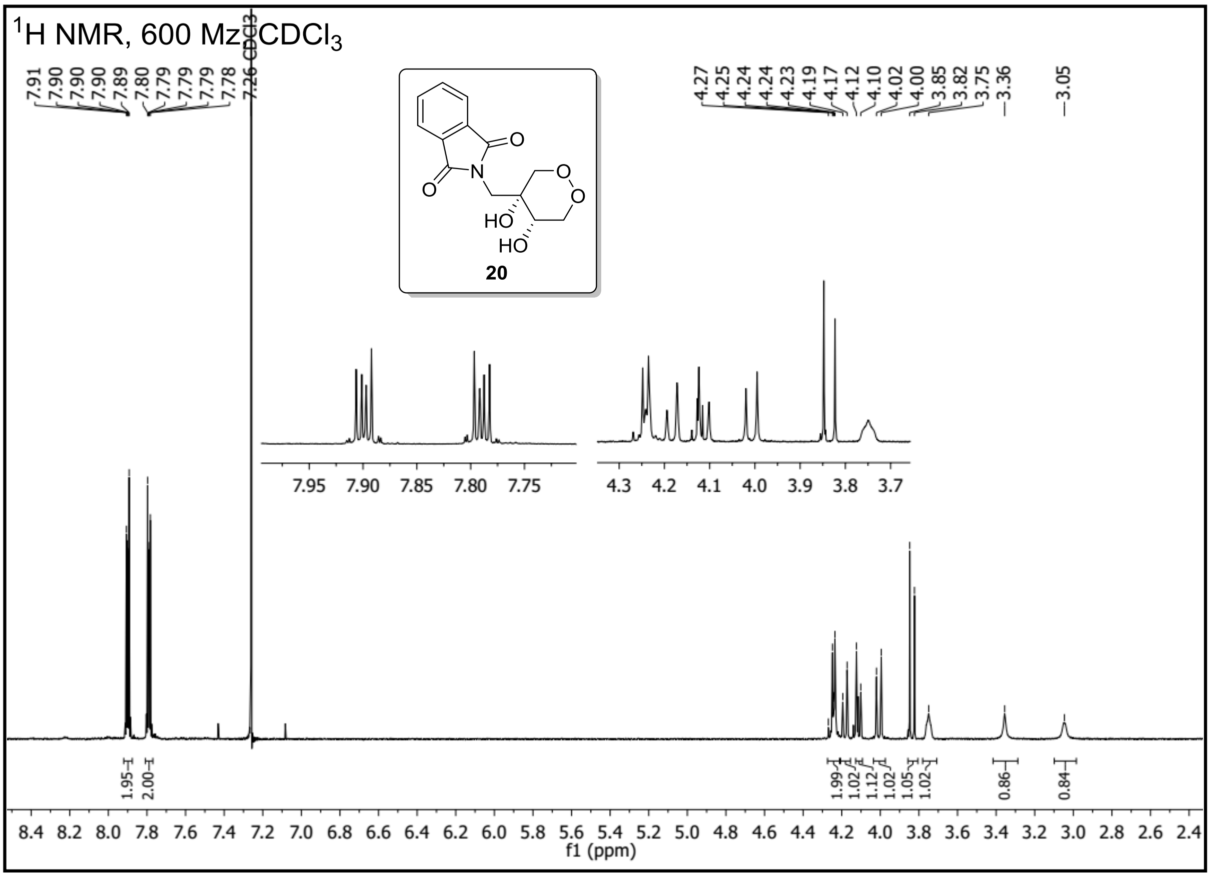

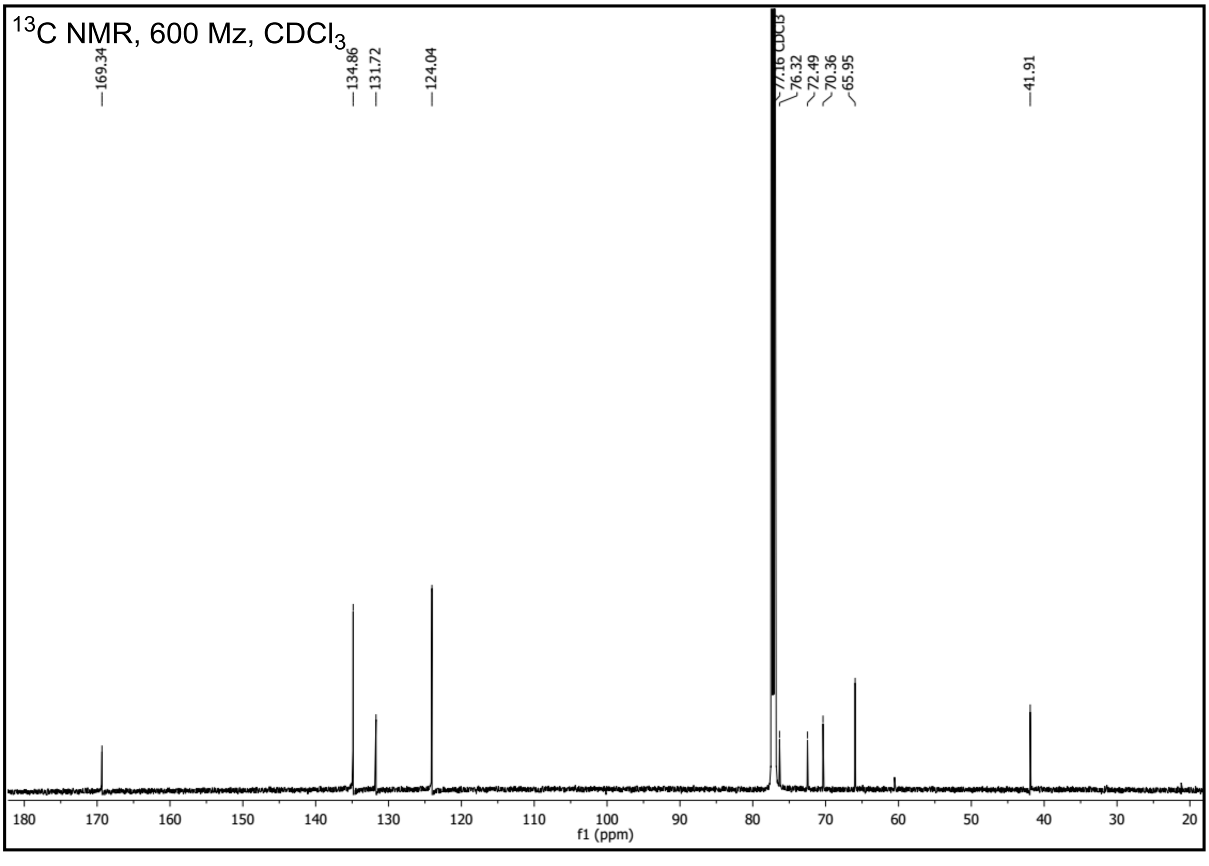

(±)-2-[2-((4*R*,5*S*)-4,5-Dihydroxy-1,2-dioxan-4-yl)ethyl]-isoindoline-1,3-dione (21)

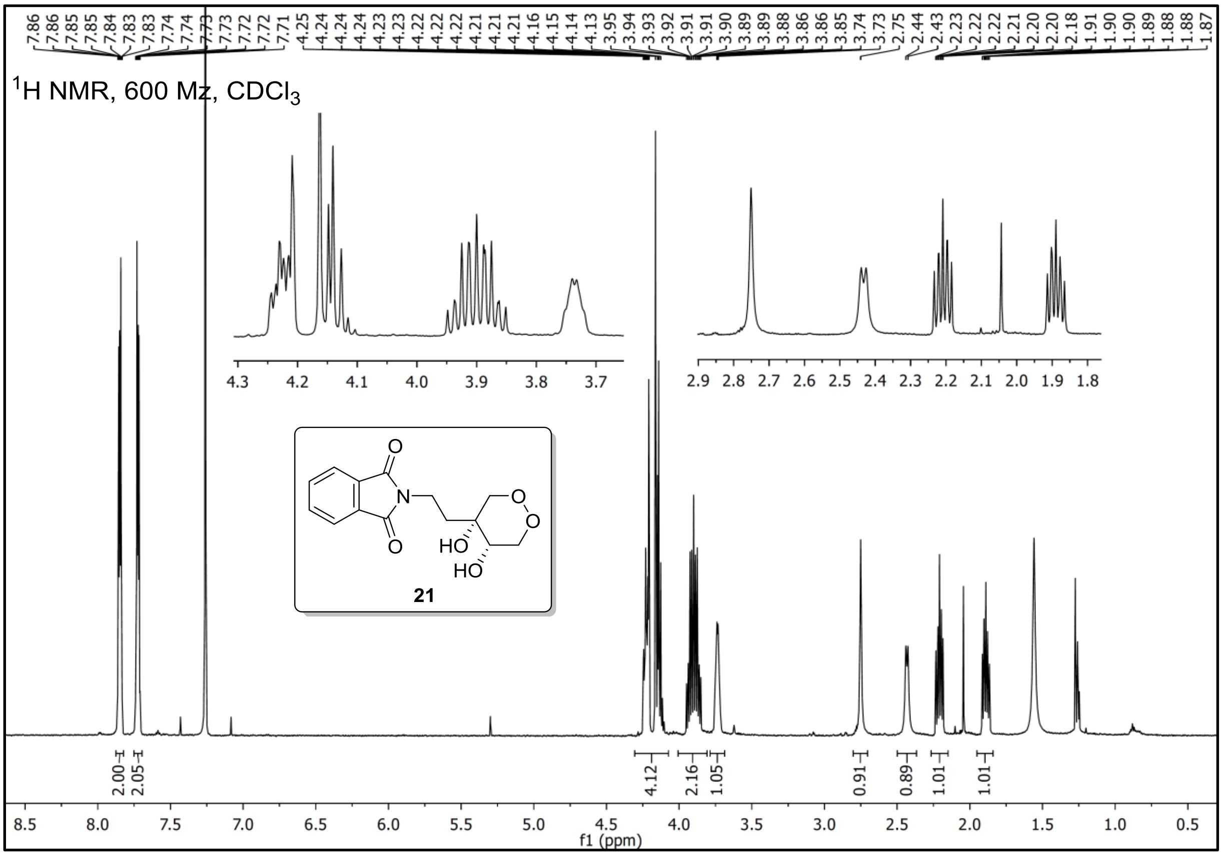

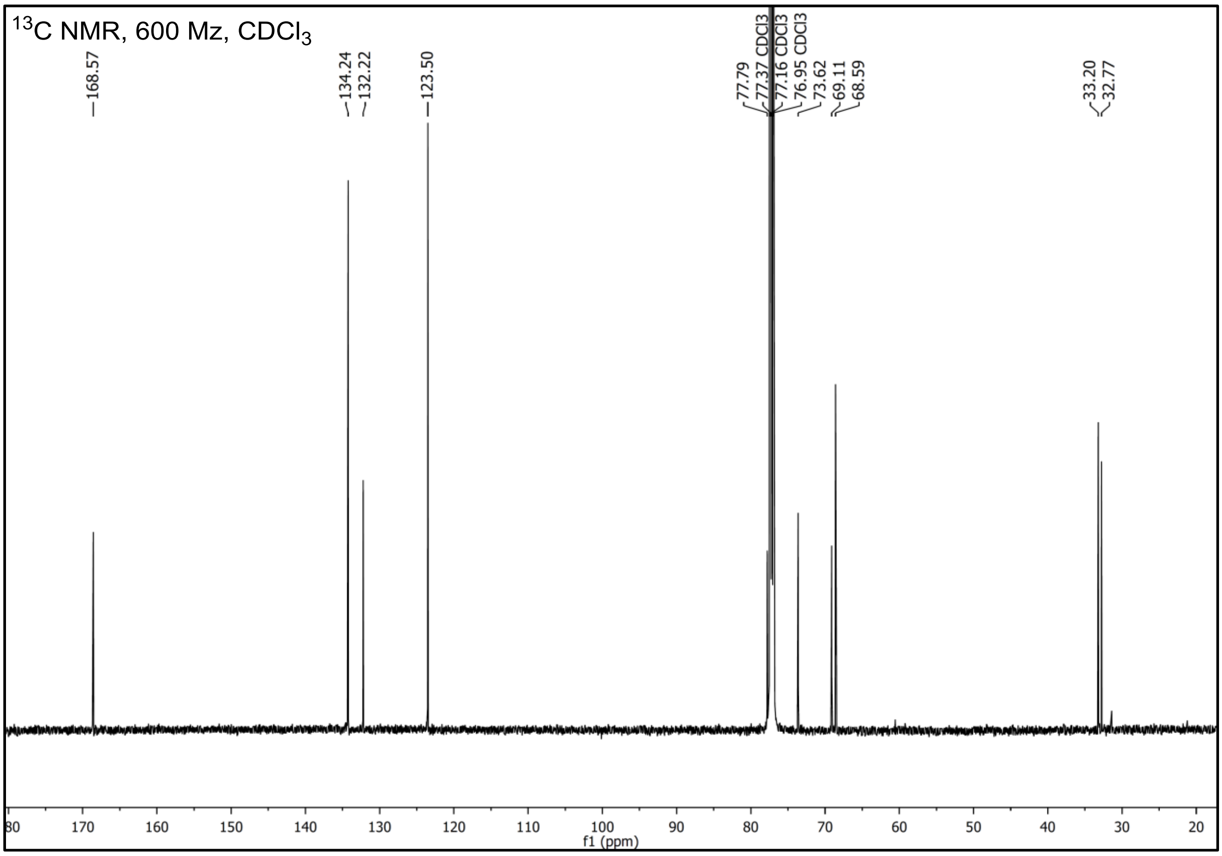

2-(((3a*R*,7a*S*)-2,2-Dimethyldihydro-[1,3]dioxolo[4,5-*d*][1,2]dioxin-3a(4*H*)-yl)methyl)isoindoline-1,3-dione (22)

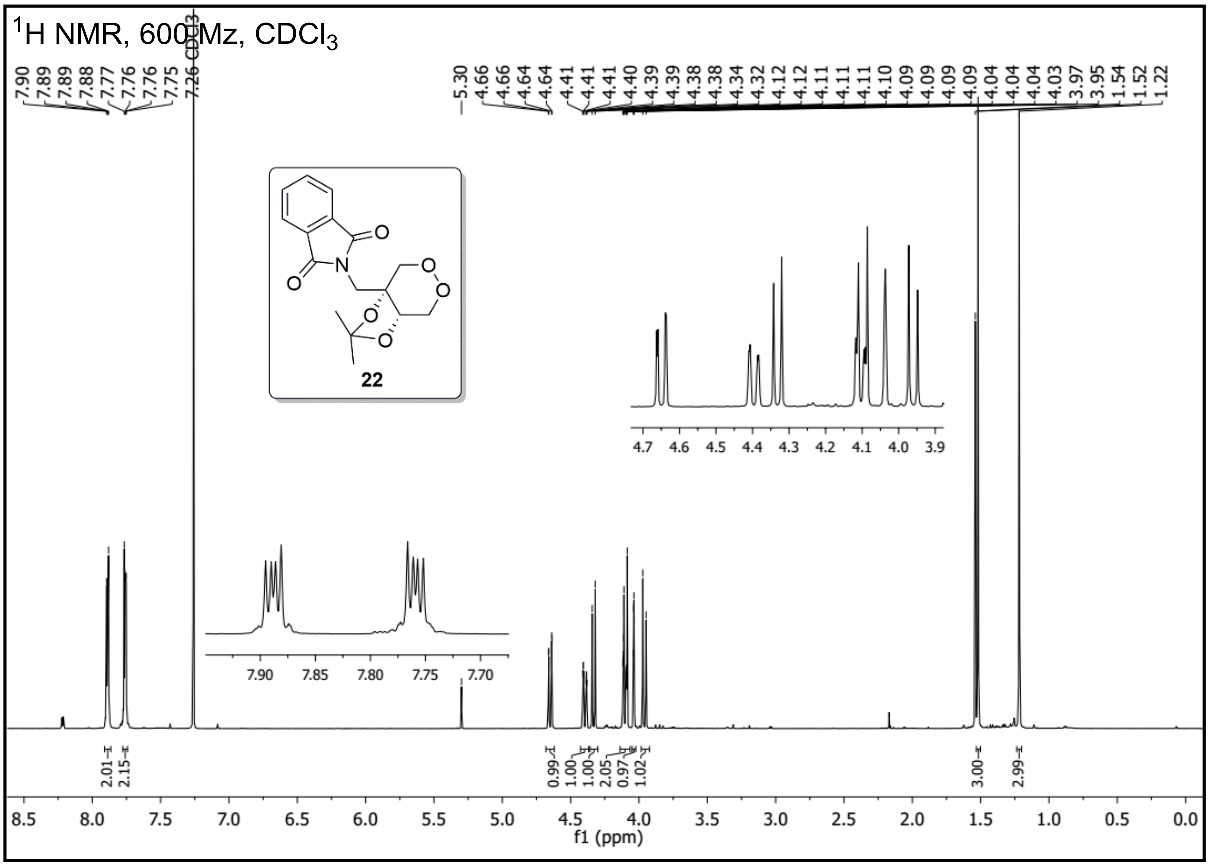

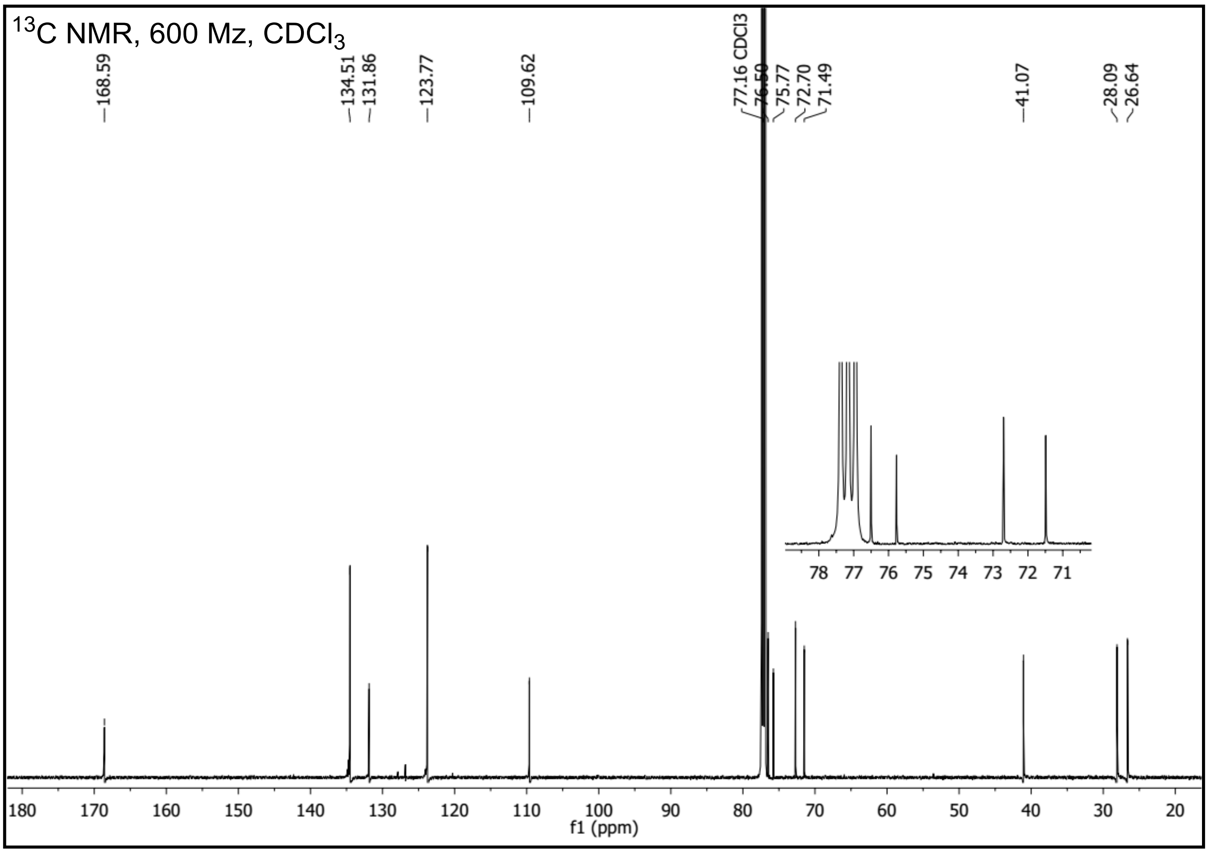

(±)-2-(2-((3a*R*,7a*S*)-2,2-Dimethyldihydro-[1,3]dioxolo[4,5-*d*][1,2]dioxin-3,a(4*H*)-yl)ethyl)isoindoline-1,3-dione (23)

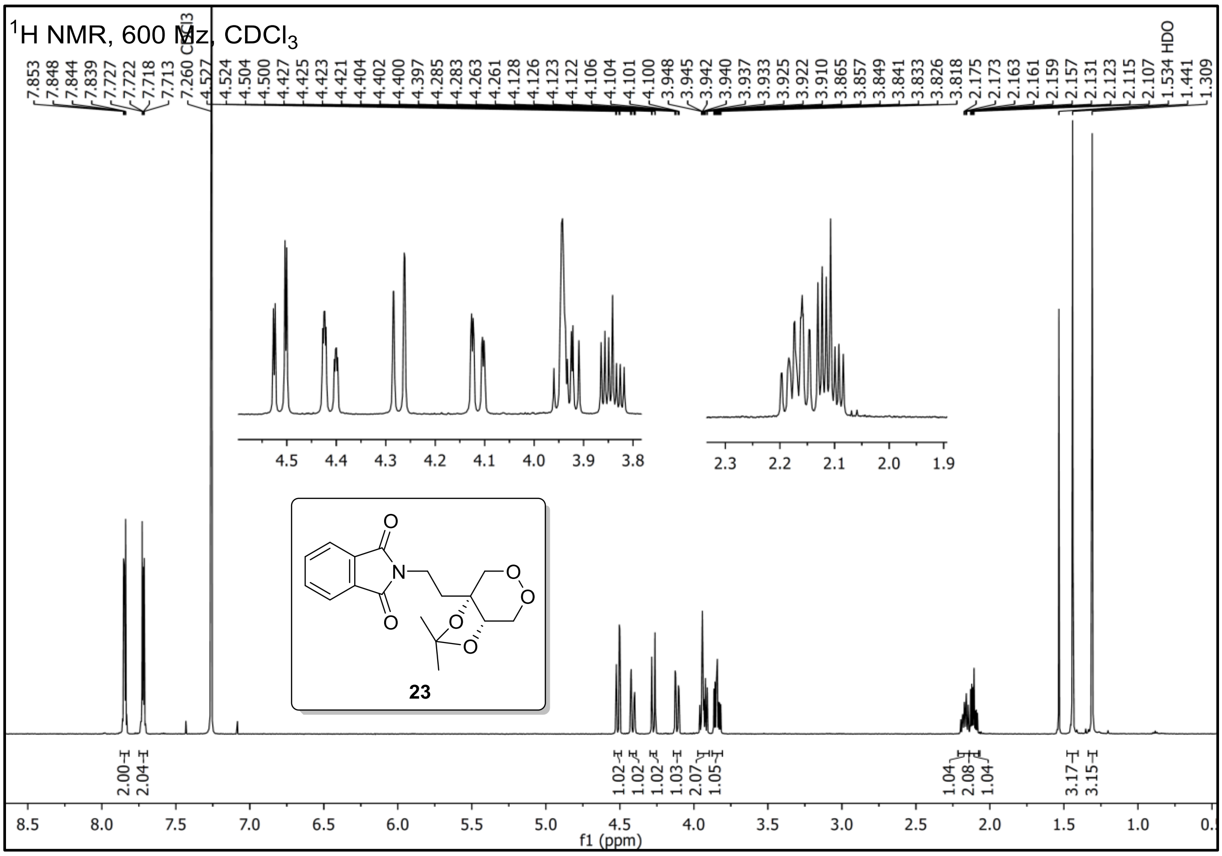

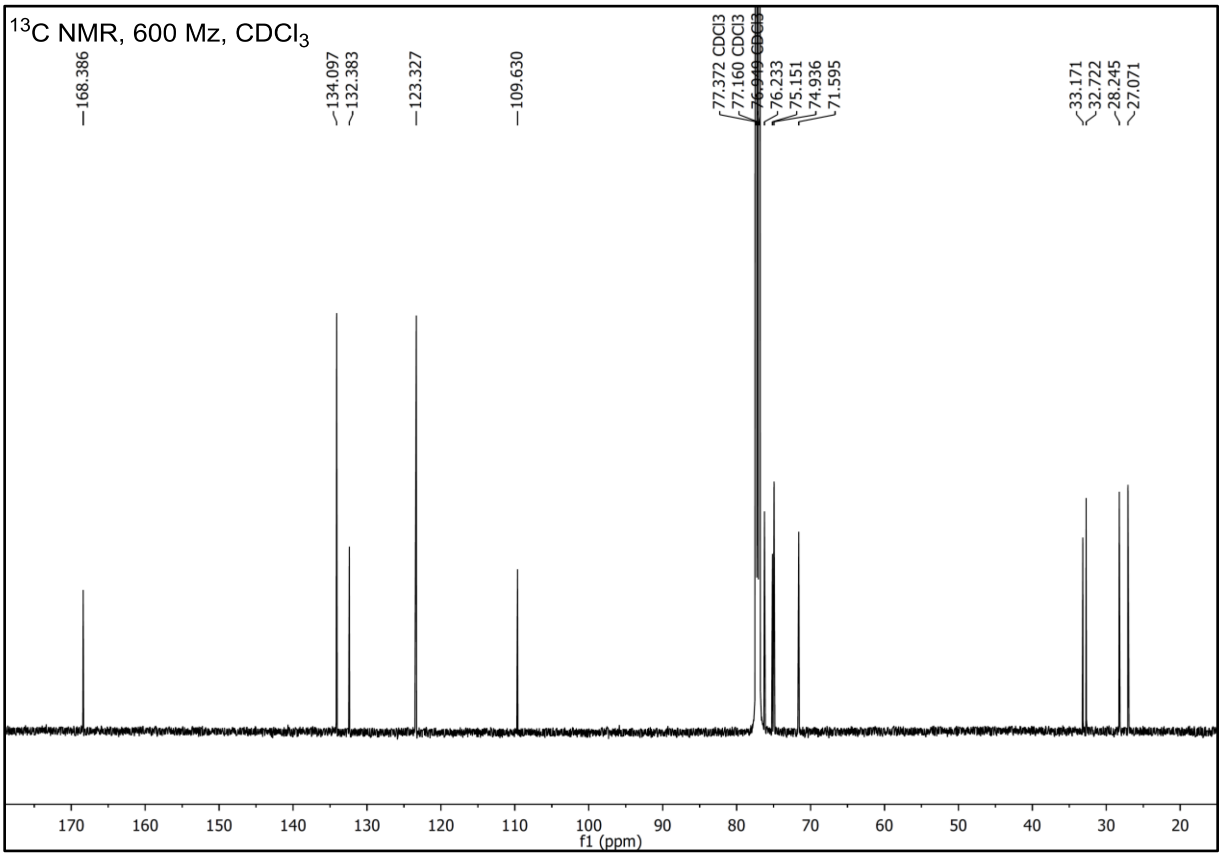

(±)-2-(2-((3*aR*,6*aS*)-2,2-Dimethyldihydrofuro[3,4-*d*][1,3]dioxol-3a(4*H*)-yl)ethyl)isoindoline-1,3-dione (24)

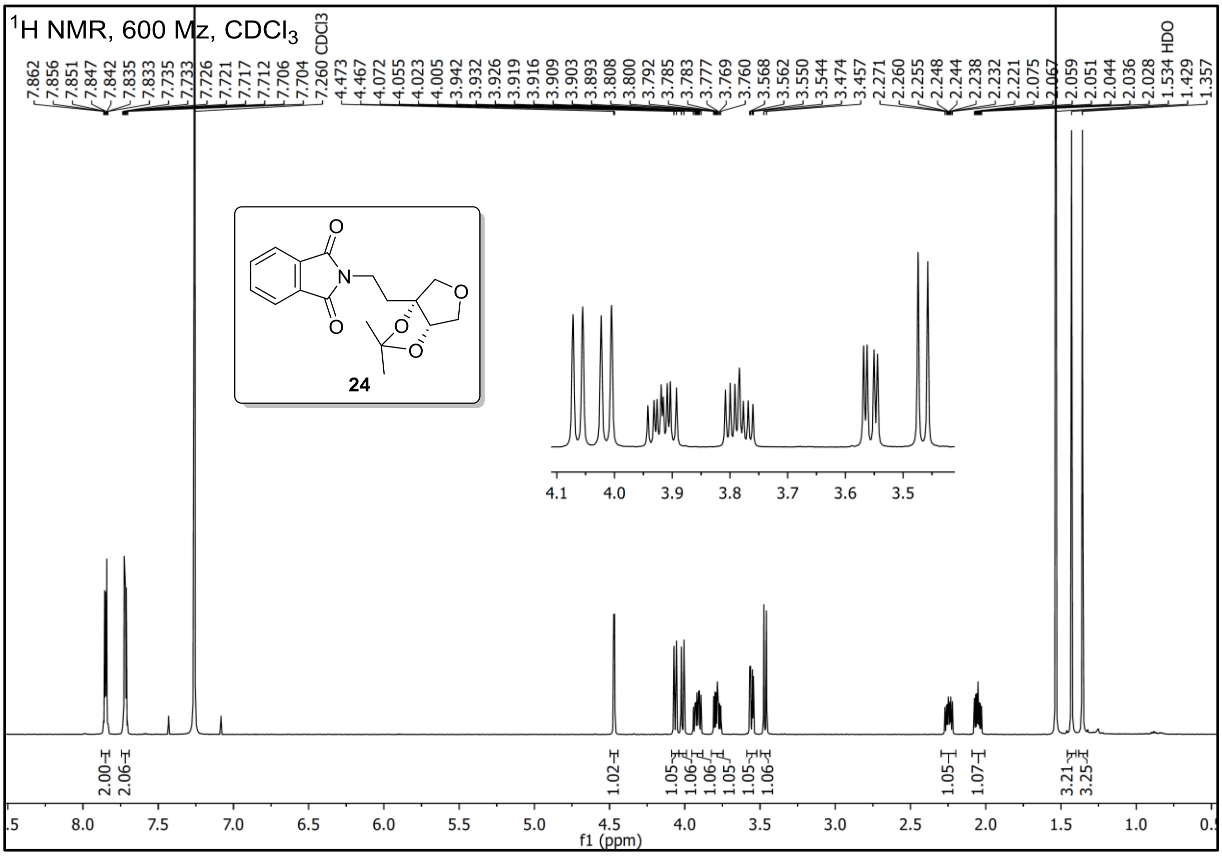

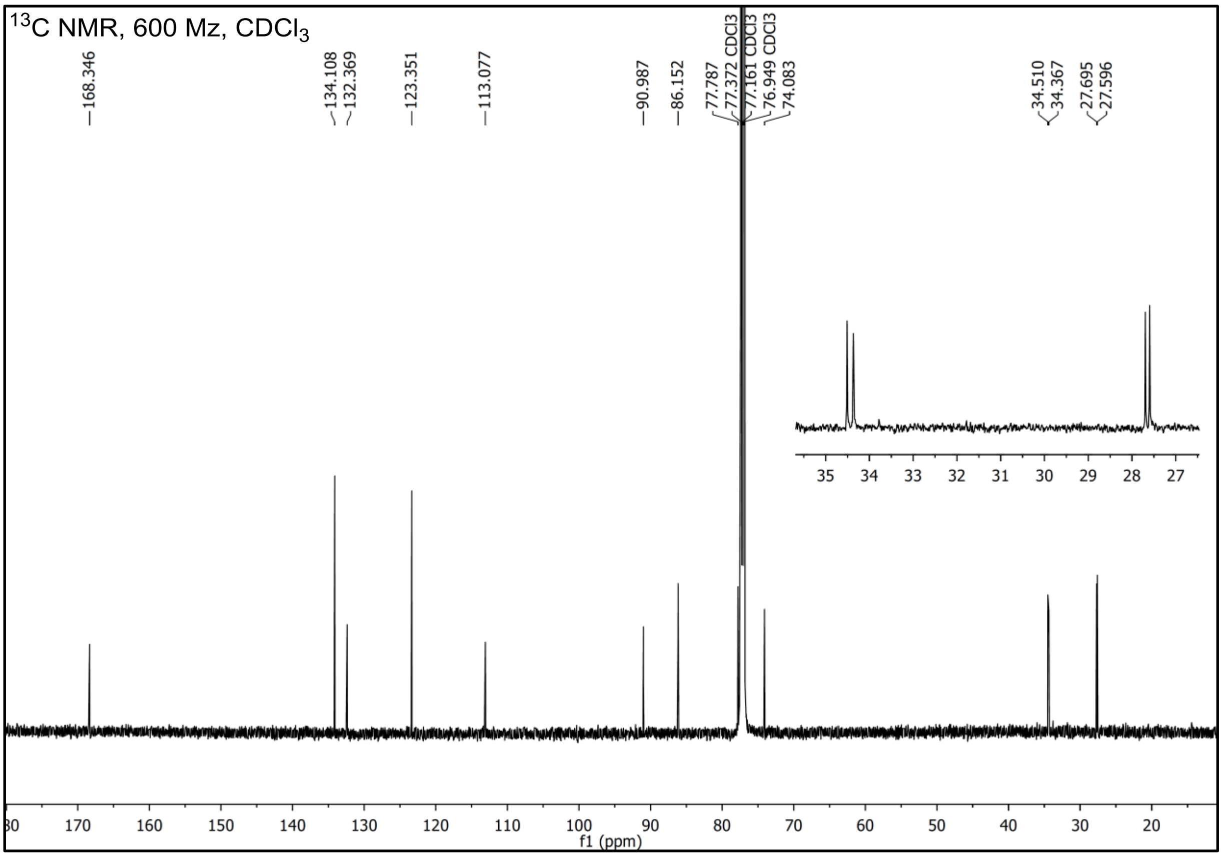

(±)-2-(2-((1*R*,6*S*)-3,4,7-Trioxabicyclo[4,1,0]heptan-1-yl)ethyl)-isoindoline-1,3-dione (25)

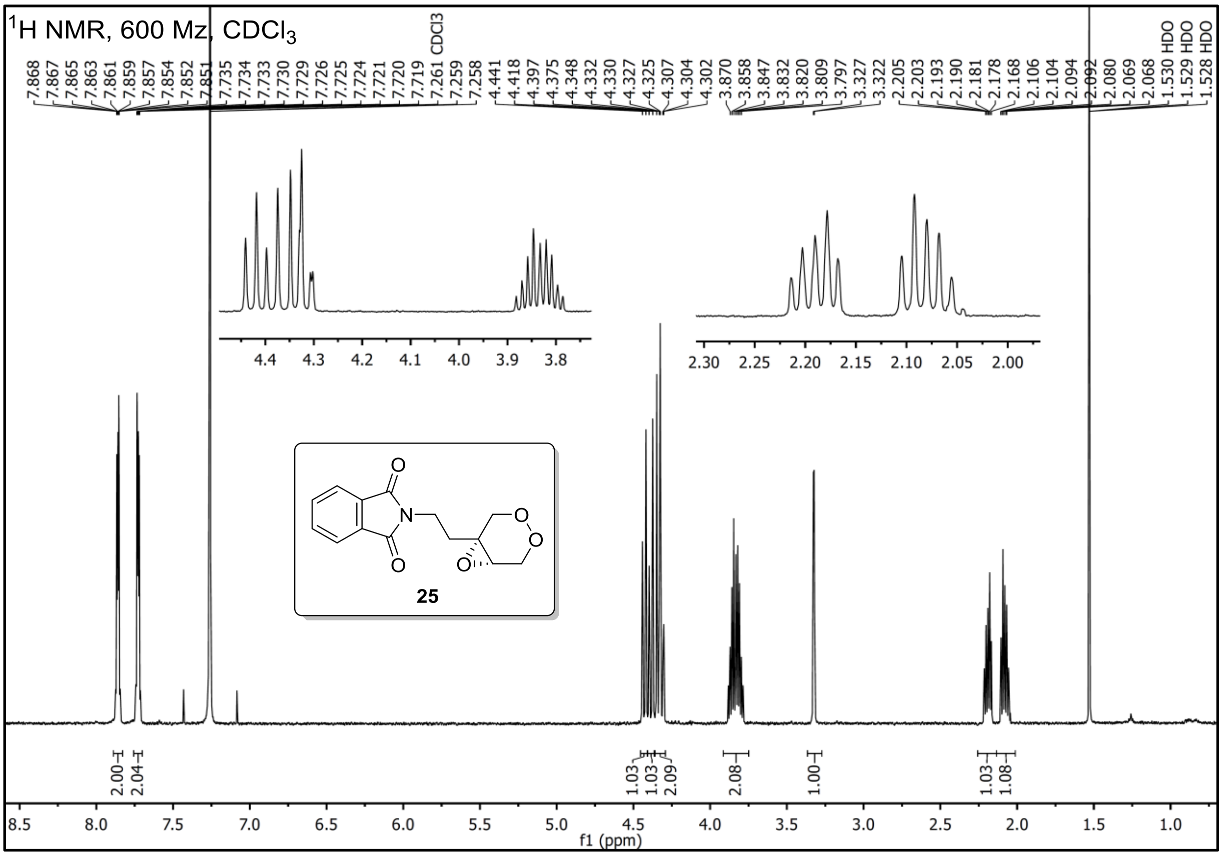

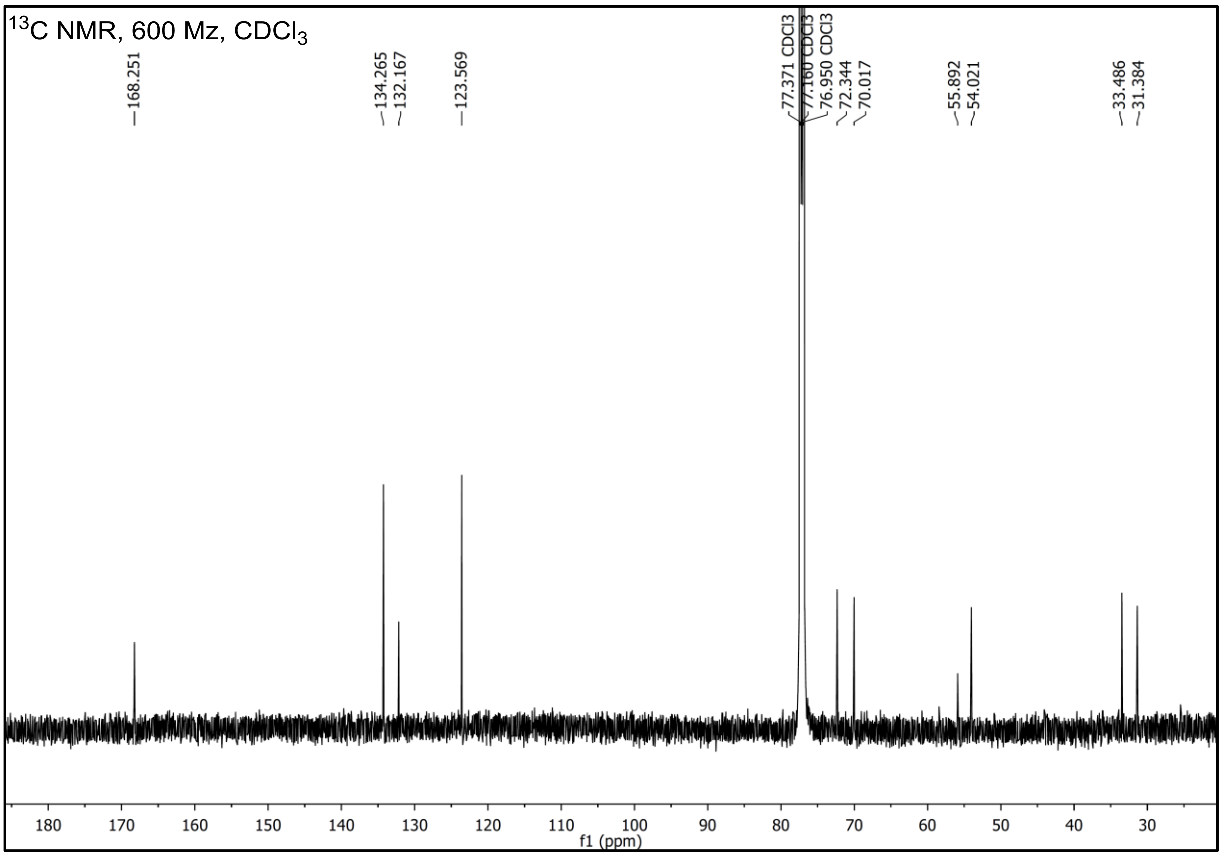

(±)-2-(2((4*R*,5*R*)-4,5-dihydroxy-1,2-dioxan-4-yl)ethyl)isoindoline-1,3-dione (26)

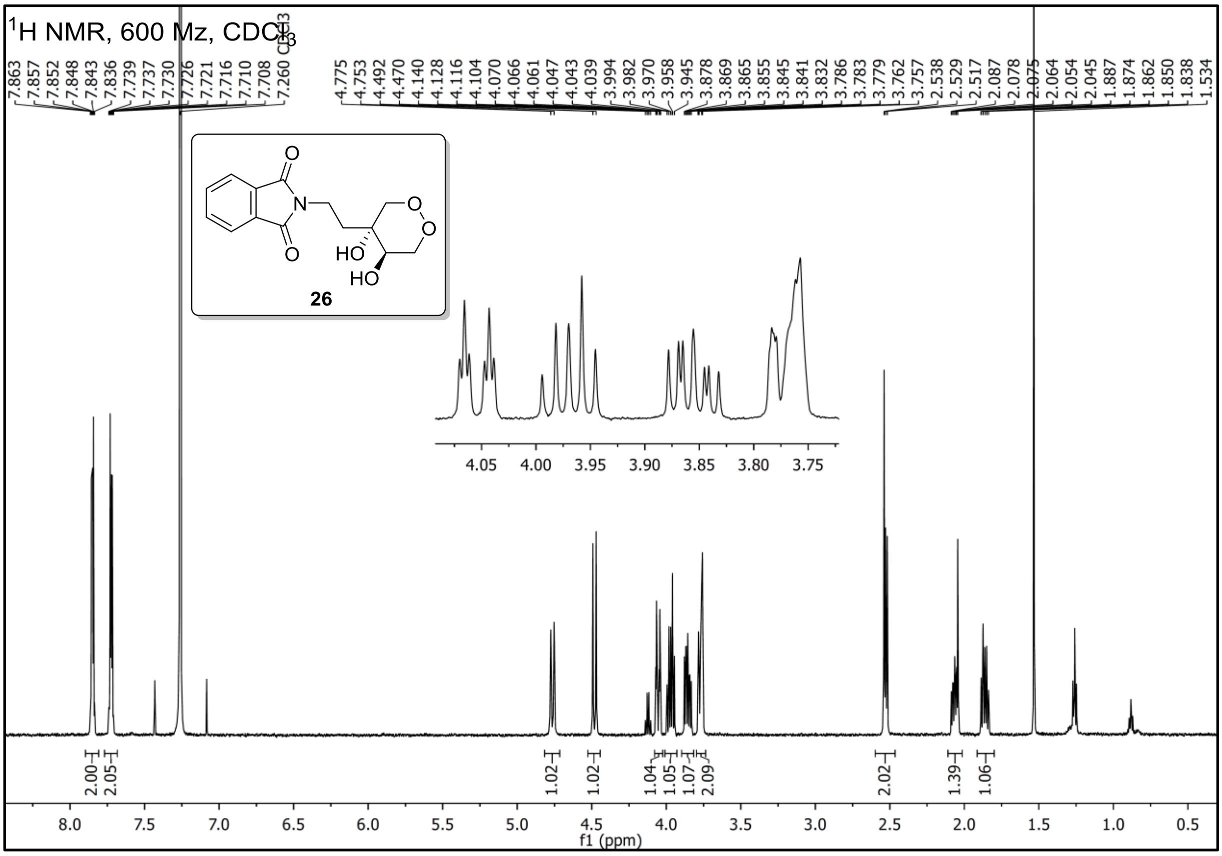

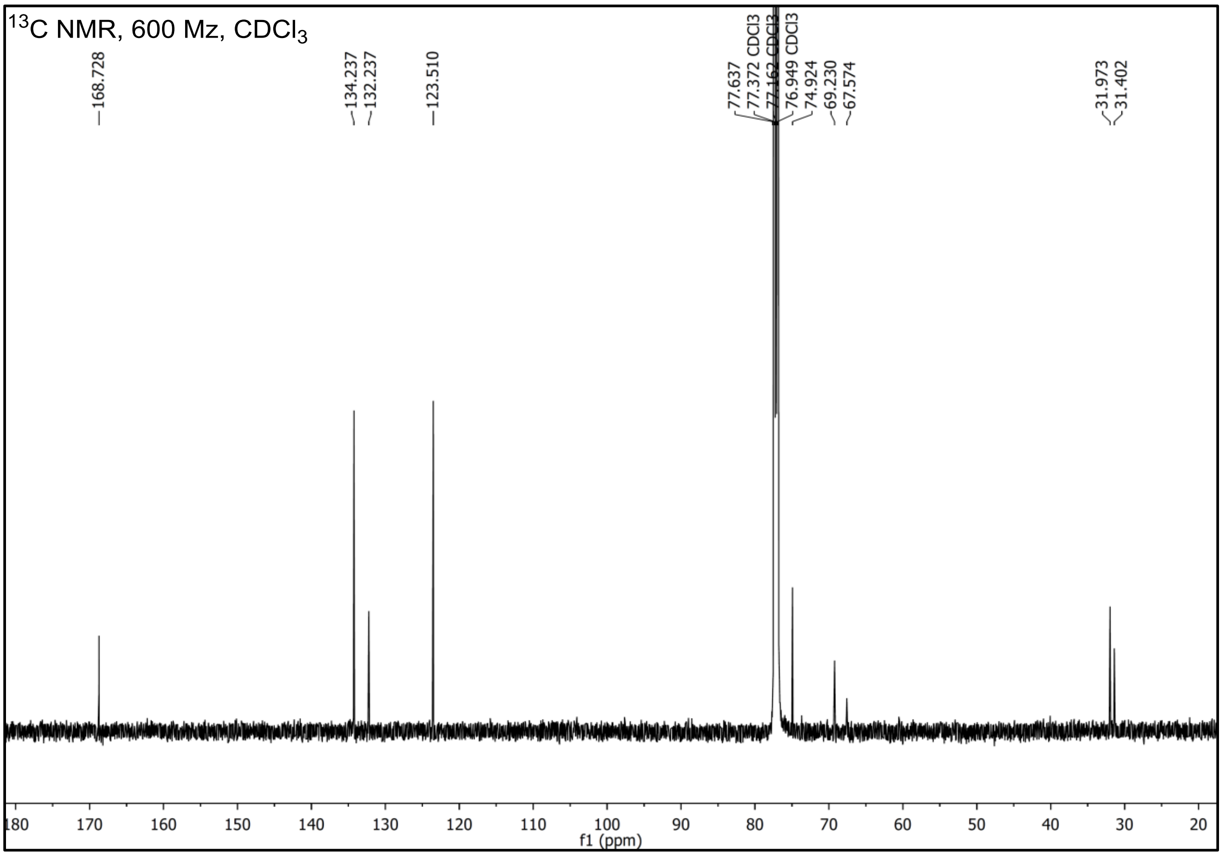

(±)-2-(2-((4*R*,5*S*)-4,5-bis(Hydroxymethyl)2-2-dimethyl-1,3-dioxolan-4-yl)ethyl)isoindoline-1,3-dione (28)

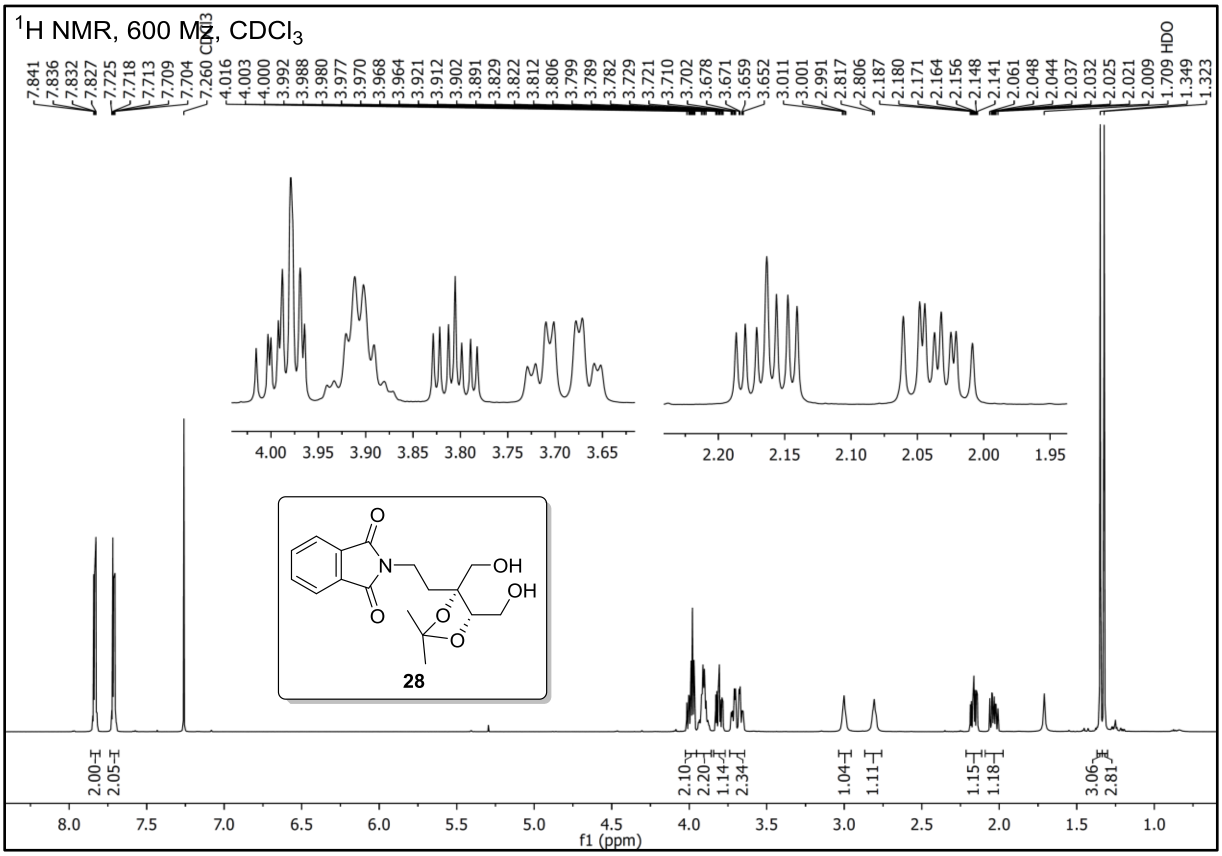

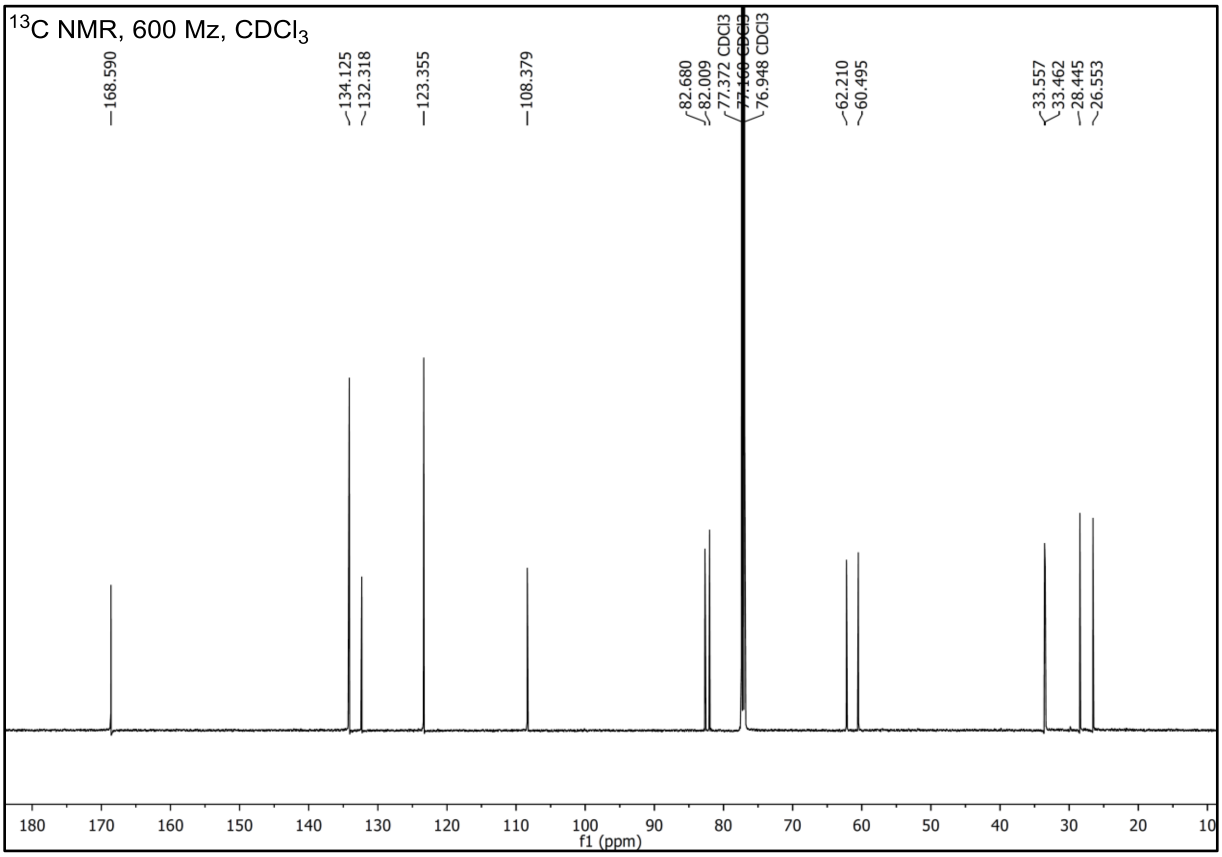

References

[1.] T. James, I. Simpson, J. A. Grant, V. Sridharan, A. Nelson, *Org.Lett.* **2013,** *15* 6094-6097.

[2.] H. Zhu, J. G. Wickenden, N. E. Campbell, J. C. T. Leung, K. M. Johnson, G. M. Sammis, *Org.Lett.* **2009,** *11* 2019-2022.

[3.] V. Rerat, G. Dive, A. A. Cordi, G. C. Tucker, R. Bareille, J. Amédé, L. Bordenave, J. Marchand-Brynaert, *J.Med.Chem.* **2009,** *52* 7029-7043.

[4.] X. Xiao, S. Antony, G. Kohlhagen, Y. Pommier, M. Cushman, *Bioorg.Med.Chem.* **2004,** *12* 5147-5160.

[5.] A. Hequet, O. N. Burchak, M. Jeanty, X. Guinchard, E. Le Pihive, L. Maigre, P. Bouhours, D. Schneider, M. Maurin, J. M. Paris, J.-N. Denis, C. Jolivalt, *ChemMedChem* **2014,** *9* 1534-1545.

[6.] F. Klepper, K. Polborn, T. Carell, *Helv.Chim.Acta* **2005,** *88* 2610-2616.

[7.] A. H. Li, S. Moro, N. Forsyth, N. Melman, X. D. Ji, K. A. Jacobson, *J.Med.Chem.* **1999,** *42* 706-721.

[8.] D. S. Pedersen, D. K. Taylor, E. R. Tiekink, *Acta Cryst.* **2007,** *E63* o4301.

[9.] G. Xiong, M. Wei, Y. Zhou, Y. Li, F. Zhang, Y. Gong, *Synthesis* **2011,** *2011* 3439-3446.

[10.] K. Lukin, V. Kishore, T. Gordon, *Org.Process Res.Dev.* **2013,** *17* 666-671.

[11.] F. Borg-Visse, F. Dawans, E. Maréchal, *J.Polym.Sci.Polym.Chem.Ed.* **1980,** *18* 2481-2489.

[12.] D. S. Pedersen, C. Rosenbohm, *Synthesis* **2001,** *2001* 2431-2434.
